# Intracellular regulation of a serotonin-gated ion channel links receptor trafficking to memory

**DOI:** 10.1016/j.isci.2026.117309

**Published:** 2026-08-25

**Authors:** Leona Cesar, Davide Zabeo, Andrea Cellini, Emelie Aspholm, Alexander Kolsrud, Dimitra Panagaki, Johanna Louise Höög, Julia Morud

**Affiliations:** 1 Department of Chemistry and Molecular Biology, University of Gothenburg, Gothenburg, Sweden

**Keywords:** *C. elegans*, synaptic plasticity, serotonin receptor, avoidance learning

## Abstract

Learning and memory arise from synaptic plasticity, the ability of neurons to modify connectivity through experience-dependent changes in receptor localization and signaling. Here, we identify a short intracellular motif within the serotonin-gated ion channel LGC-50 that links molecular receptor dynamics to behavioral plasticity in *Caenorhabditis elegans*. Deletion of residues 363–379 in the intracellular M3-4 loop caused receptor clustering in intracellular compartments and abolished learning-induced redistribution without altering receptor function or immediate memory recall. Interestingly, animals expressing the truncated receptor displayed impaired retrieval of aversive memories 1 h after training, revealing a role for receptor trafficking in memory stability. Combining molecular, ultrastructural, and behavioral analyses *in vivo*, we show how intracellular receptor motifs govern experience-dependent plasticity. These findings demonstrate that precise receptor localization and trafficking shape neural circuit adaptation and reveal a conserved mechanism by which receptor dynamics support the persistence and retrieval of memory across species.

## Highlights

• LGC-50 intracellular motif controls activity-dependent receptor trafficking
• Disrupted LGC-50 trafficking impairs memory retrieval but not learning
• Receptor localization links molecular trafficking to behavioral plasticity

## Graphical abstract

**Figure:**
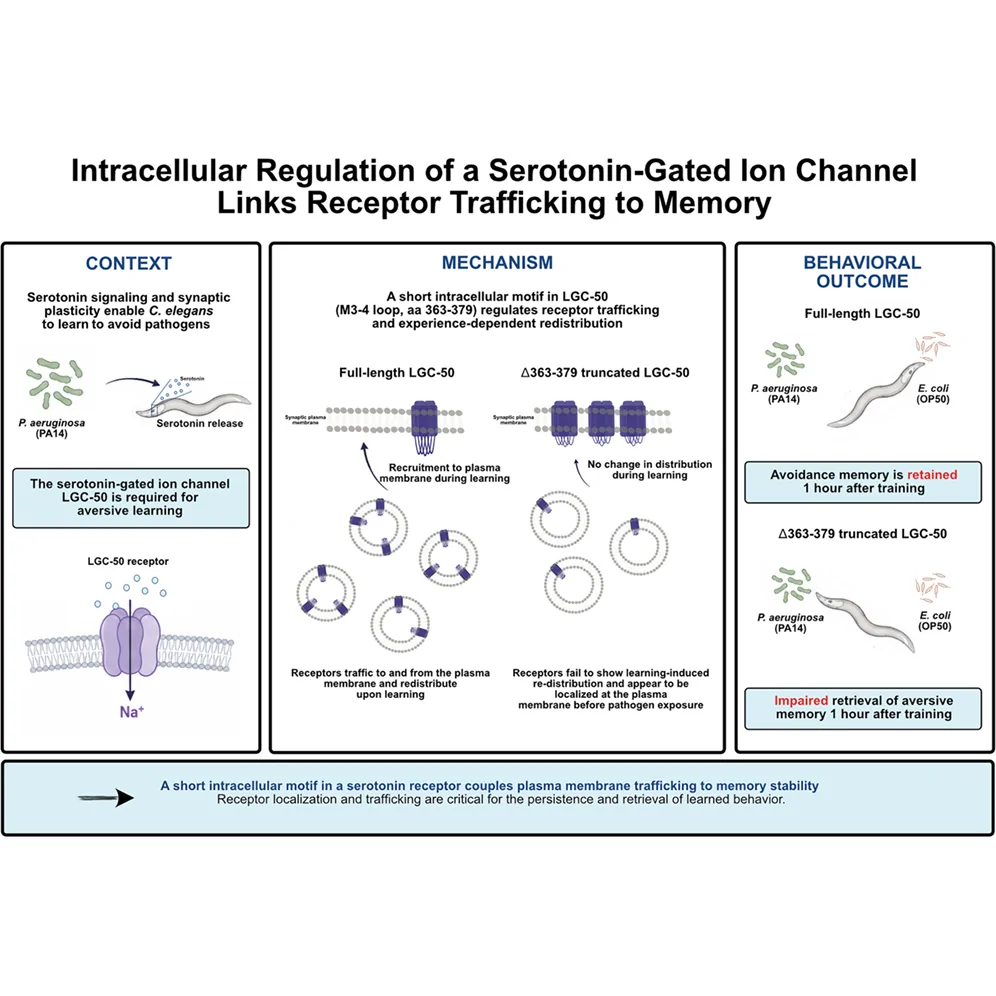

## Teaser

Neuroscience; Cell biology

## Introduction

Nervous systems possess an incredible capacity to adapt information processing over time, integrating sensory input with evolving internal states to enable experience-dependent decisions about when and how to act. This phenomenon, broadly referred to as neuronal plasticity, forms the basis for essential higher-order processes such as learning, memory formation, and behavioral adaptation.^1^^,^^2^^,^^3^ At the cellular level, plasticity is thought to emerge primarily from activity-dependent modifications in neuronal excitability within interconnected neural circuits.^4^^,^^5^ These modifications can occur over a wide range of timescales, from rapid millisecond changes to long-lasting structural and molecular alterations that persist for days or longer.^6^ A key mechanism driving such shifts in excitability involves the regulation of synaptic receptor abundance, particularly changes in the number and composition of ligand-gated ion channels (LGICs) at the postsynaptic membrane. Of special importance are the excitatory glutamate-gated receptors, including α-amino-3-hydroxy-5-methyl-4-isoxazolepropionic acid (AMPA) and N-methyl-D-aspartate (NMDA) receptors,^7^ which mediate fast excitatory neurotransmission. In contrast, the inhibitory gamma-aminobutyric acid A (GABA_A_) receptor provides a counterbalancing inhibitory influence on network activity.^8^ By dynamically adjusting their abundance and distribution, these receptors recalibrate circuit responsiveness, enabling the brain to encode experience and adapt. The precise mechanisms governing these dynamic changes in receptor abundance remain poorly understood.

The trafficking of LGICs to the plasma membrane and their incorporation into synapses are tightly regulated through interactions with specific regulatory proteins.^9^^,^^10^^,^^11^^,^^12^ Many of these mechanisms are activity-dependent, linking neuronal activation to plasma membrane trafficking and thereby influencing processes such as memory formation.^1^^,^^13^^,^^14^ Research to date has largely highlighted events proximal to the synapse, including plasma membrane trafficking of receptors, endosomal recycling, and their anchoring by structural organizer proteins.^10^^,^^15^^,^^16^ Extensive work in mammalian systems, particularly on AMPA receptor trafficking, has established key principles governing activity-dependent receptor insertion, recycling, and synaptic stabilization using both *in vitro* and *in vivo* approaches.^17^^,^^18^^,^^19^ However, how analogous trafficking mechanisms operate in intact circuits *in vivo* and across diverse LGIC families remains less well understood. Within NMDA, AMPA, and GABA_A_ receptors, distinct regulatory motifs have been identified as being critical for proper localization to the plasma membrane and synapses.^20^^,^^21^^,^^22^^,^^23^ However, the difficulty of tracking receptors *in vivo* means that dissecting these mechanisms at a molecular level in intact mammalian systems remains more challenging. The nematode *Caenorhabditis elegans* (*C. elegans*) provides a powerful model, owing to its compact nervous system and lifelong transparency, which facilitate live imaging and genetic manipulation.^24^^,^^25^^,^^26^ In addition, *C. elegans* carries genomic homologs of human LGICs as well as critical components of synaptic plasticity, such as scaffolding, anchoring, signaling, and adhesion proteins, underscoring its utility as a model system for studying the molecular basis of synaptic plasticity.^27^^,^^28^^,^^29^^,^^30^^,^^31^ Yet the precise roles of these proteins in *C. elegans* and whether regulatory motifs function analogously to those in mammals are not well known.

**Figure 1:**
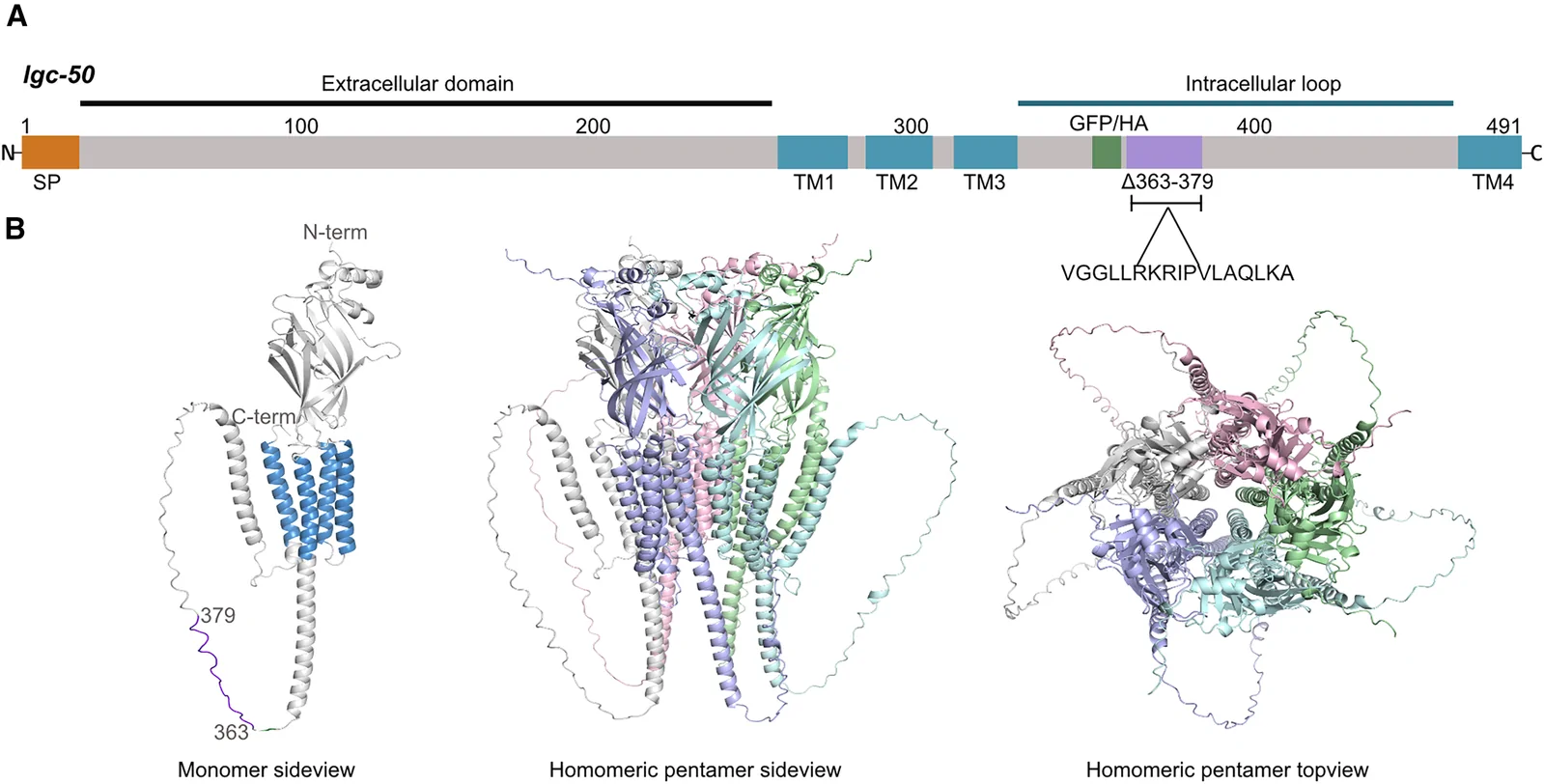
LGC-50 gene and protein representation (A) Graphical example of *lgc-50* indicating location of the predicted signaling peptide (SP), CRISPR insertion site for GFP and the position of residues 363–379 in the transmembrane (TM) 3-4 intracellular loop. (B) AlphaFold3^37^^,^^38^ sideview and top view 3D models of an LGC-50 protein monomer and homomeric pentamer, with residues 363–379 highlighted in the monomer model (purple).

*C. elegans* inhabits a complex microbial environment and must learn to discriminate between odors, associating them with either positive reinforcers, such as the presence of food, or negative ones, such as pathogenic bacteria.^32^^,^^33^^,^^34^ These learning abilities allow short-term memory to be assessed in *C. elegans* by exposing animals to pathogenic bacteria, such as the *Pseudomonas aeruginosa* strain PA14, and subsequently testing their acquired avoidance of them.^34^^,^^35^ Our recent work identified the homomeric cationic serotonin-gated channel LGC-50 as a critical component of serotonin-dependent pathogen avoidance learning.^36^ A large intracellular domain in the loop between transmembrane (TM) helices 3 and 4 (the M3-4 loop) was shown to be important for plasma membrane localization in *Xenopus laevis* oocytes expressing *lgc-50.* Truncation of residues 363–379 in the M3-4 loop (Figures 1A and 1B) increased the serotonin-induced current by approximately 1,000-fold, suggesting enrichment of the channel on the plasma membrane.^36^ In *C. elegans*, exposure of worms to PA14 bacteria caused LGC-50 to redistribute, increasing its intensity in the nerve ring. The receptor was found to play a critical role during signal integration in the interneuron RIA, underlying aversive associative learning to the pathogen PA14. However, the intracellular origin of this redistribution remains unclear and whether disruption of this process affects pathogen avoidance learning has yet to be determined.

Here, we describe the function of the intracellular motif in LGC-50 that regulates the receptor’s subcellular localization, using high-resolution electron and light microscopy. Truncation of this motif causes the receptor to form pronounced clusters at cellular membranes, independent of prior learning experience. Moreover, constant retention of the receptor at the plasma membrane interferes with activity-dependent trafficking, thereby impairing memory retrieval despite normal learning. This impairment is further reflected in the failure of the truncated receptor to undergo learning-induced changes in clustering, in contrast to the significant redistribution observed in control animals.

## Results

### An intracellular motif in LGC-50 regulates localization and receptor clustering

**Figure 2:**
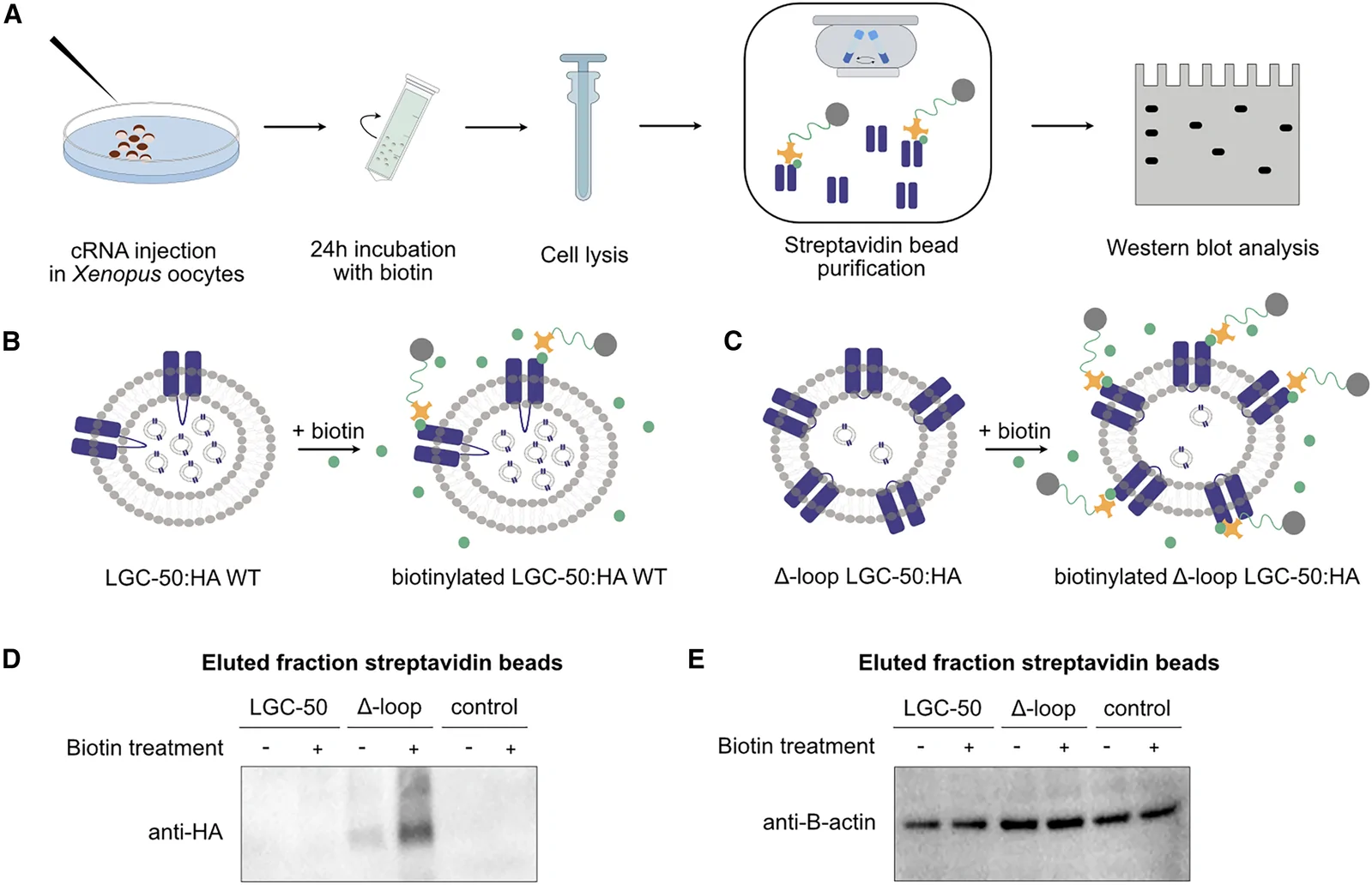
Higher amounts of surface-localized LGC-50 upon truncation of the 363–379 residues (A) Graphical representation of the biotinylation experiment workflow. (B and C) Graphical description over the two scenarios showing differences in receptor membrane localization between full-length LGC-50 and LGC-50:GFPΔ363–379. (D) Western blot analysis using an anti-HA antibody on eluted fractions after treatment with none-membrane permeable biotin shows that higher protein quantity from LGC-50:GFPΔ363–379:HA after streptavidin bead purification compared to the full-length protein LGC-50:HA. (E) Western blot analysis using anti-B-actin indicates even sample loading across genotypes.

Having previously observed that LGC-50 current size in a *Xenopus* heterologous system was influenced by a small truncation of the M3-4 loop (LGC-50Δ363–379, Figures 1A and 1B),^36^ we investigated enrichment of LGC-50 protein on the plasma membrane of *Xenopus* oocytes. To enable detection of the protein via western blot assay, we inserted an HA-tag after codon G360 into the full length LGC-50 and the truncated LGC-50Δ363–379 constructs (Figure 1A). The HA-tag did not interfere with protein function as validated by two-electrode voltage clamp (supporting Figure 1A). Oocytes expressing the full length LGC-50:HA or the truncated LGC-50Δ363–379:HA construct were treated with a membrane-impermeable biotin reagent and prepared for subsequent lysis, streptavidin affinity purification, and western blot analysis (Figures 2A–2C, supporting Figure 1). LGC-50 protein was detected in the biotinylated fraction of both samples using an anti-HA antibody; however, the signal detected in the sample expressing the truncated construct appeared to be higher than the full-length control (Figure 2D, supporting Figures 1B–1D). To account for this variation in baseline expression, we normalized the biotinylated surface expression from three individual replicates to the whole-cell LGC-50 expression. This suggests plasma membrane-localized LGC-50 to be higher in the truncated form of LGC-50 in all three replicates compared to the full-length construct (supporting Figures 1E and 1F). The total amount of protein in each sample was validated using anti-B-actin, which showed that protein content in each sample did not differ (Figure 2E). We thus conclude that the current amplitude difference that we previously observed in electrophysiology experiments is indeed caused by an enrichment of LGC-50 protein on the plasma membrane.

We then sought to determine the effect of this truncation *in vivo*. For this purpose, we used a CRISPR-edited strain with GFP inserted into the M3-4 loop of *lgc-50* (Figure 1A), this insertion site was previously validated to not interfere with LGC-50’s function.^36^ In addition, we generated a deletion of residues 363–379 in *lgc-50* using CRISPR on the GFP background (Figures 1A and 1B).

To determine whether truncation of the receptor, LGC-50Δ363–379, causes alteration in the intracellular distribution or clustering of the protein in naive animals, light microscopy and ultrastructural examination with immunoelectron microscopy (immuno-EM) were employed.

**Figure 3:**
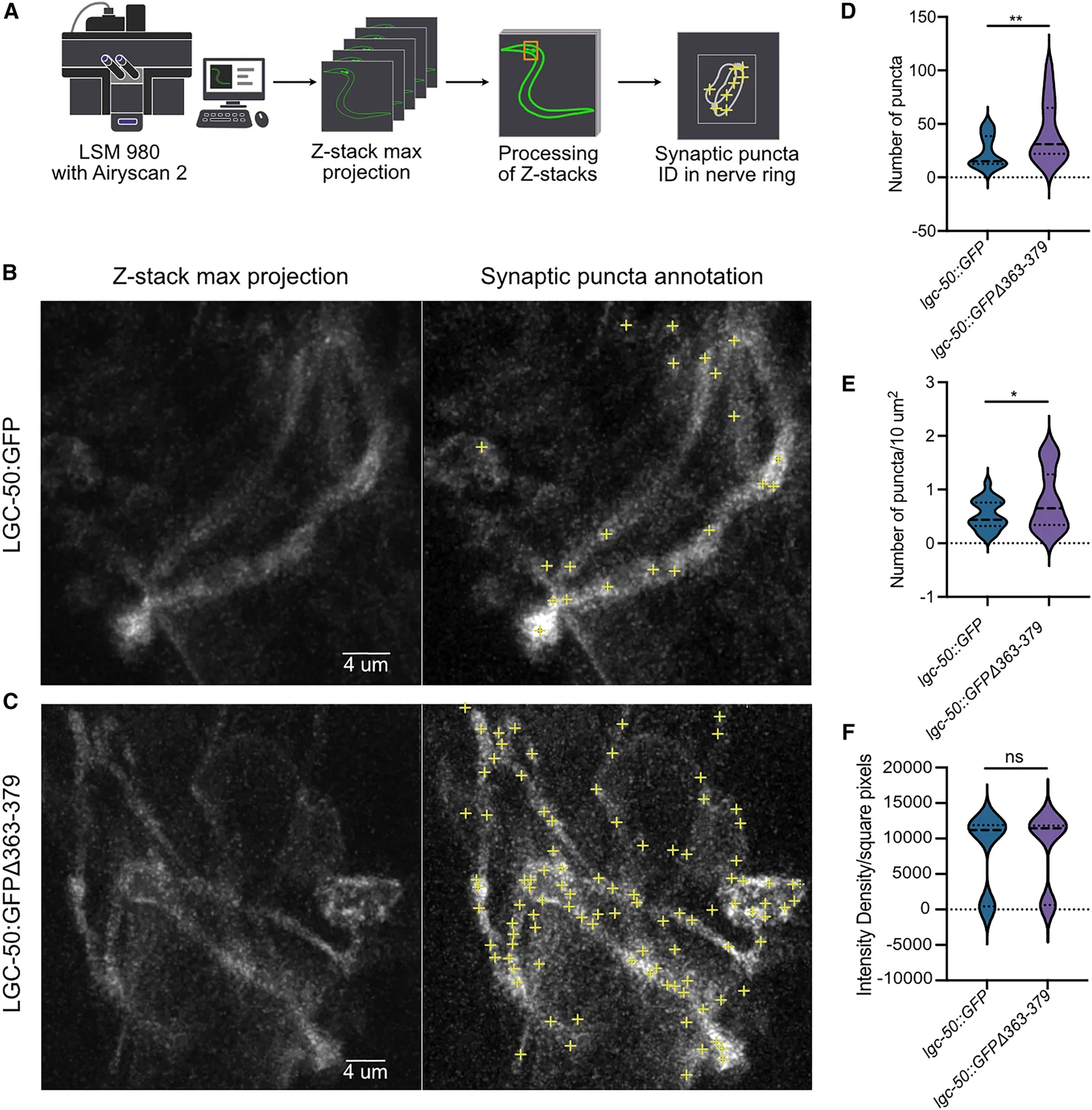
Deletion of residues 363–379 in LGC-50 significantly increases fluorescent puncta (A) Flowchart describing the work process for puncta quantification. (B) Example image of GFP signal and puncta annotation in the nerve ring in full-length *lgc-50::GFP* (*Ij120*)*.* (C) Example image of GFP signal and the puncta annotation in the nerve ring in *lgc-50::GFPΔ363–379* (*syb3377*). (D) Average total number of GFP puncta detected/worm in the nerve ring. (E) Average number of puncta in the nerve ring normalized by area/worm. (F) Total fluorescent intensity detected in the nerve ring. *N* = 21 worms LGC-50:GFP, *N* = 19 worms LGC-50Δ363–379, presented as a violin plot with median, 1^st^ and 3^rd^ quartile marked with dashed lines, ∗*p* < 0.05, ∗∗*p* < 0.005; ns, no significant difference, calculated using paired *t* test.

We performed fluorescence microscopy in both the GFP-tagged *lgc-50* strain and the *lgc-50::GFPΔ363–379* strain (Figure 3A). Since the goal was to determine whether receptors were differently localized in areas relevant for aversive olfactory learning,^34^ we focused our analysis on the nerve ring and the identification of fluorescent puncta. To enable unbiased quantification of fluorescent puncta, images were processed using a semi-automated image analysis pipeline.^39^^,^^40^ The fluorescence imaging analysis revealed that the 363–379 truncation led to an increase in fluorescent puncta, both in total number and density (puncta/μm^2^), as compared to the full-length receptor (Figures 3B–3E). These findings indicate that deletion of residues 363–379 alters the subcellular distribution and clustering behavior of LGC-50 *in vivo*. However, due to the resolution limits of fluorescence microscopy, the observed puncta could not be definitively assigned to plasma membrane, synaptic, or intracellular compartments. To ensure that this increase did not arise from higher overall receptor expression in the LGC-50:GFPΔ363–379 strain, total fluorescence intensity within the nerve ring was measured, revealing no significant difference between the two strains (Figure 3F).

**Figure 4:**
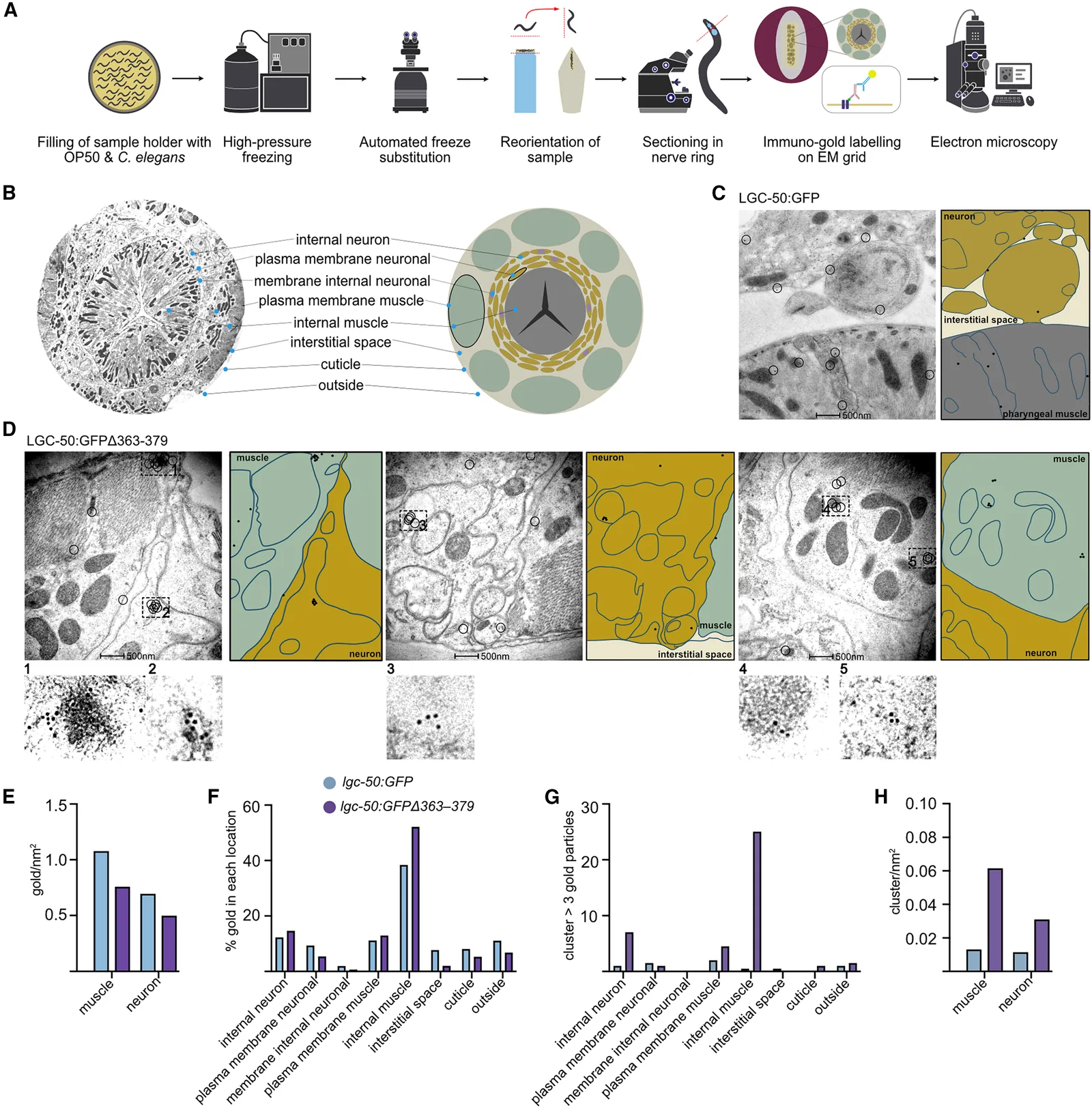
LGC-50:GFPΔ363–379 is localized in clusters in *C. elegans* (A) Graphical description of immuno-EM workflow. (B) Cross section and cartoon of *C. elegans* cross-section through the nerve ring, describing where the different tissues and cellular structures quantified in the immuno-EM are identified. (C) Micrographs and graphical description of gold-labeling in samples from full-length *lgc-50::GFP* (*Ij120*) animals. (D) Micrographs and graphical description of gold-labeling in samples from truncated *lgc-50::GFPΔ363–379* (*syb3377*) animals that indicate high incidence of gold particles in clusters. (E) Total number of gold particles per nm^2^ in muscles and neurons. (F) Distribution of gold particles in different cellular locations. (G) Number of clusters with more than three gold particles in different areas of the worm. (H) Amount of clustered gold particles normalized by area. *N* = 2 for each strain from which several micrographs were collected.

To further resolve the localization of these receptor clusters at higher spatial resolution, we next performed immuno-EM against a GFP tag.^41^^,^^42^ For optimal ultrastructural preservation, worms expressing GFP-tagged LGC-50 were high-pressure frozen, embedded in resin, and sectioned ultra-thin (Figure 4A). The GFP-tagged LGC-50 receptors, with or without truncation, were detected using gold-conjugated secondary antibodies. We compared the localization and total number of gold particles per area across different tissues and cellular structures in the nerve ring of the worm (supporting Table S1, supporting Figure 2A). As *lgc-50* was previously shown to be expressed in both nervous and muscle tissue,^36^ we focused our investigation on these tissues.

Interestingly, although the overall number of gold-labeled LGC-50 did not differ drastically between muscle or nervous tissue in the two strains (Figures 4H and 4I), the Δ363–379 deletion strain displayed a markedly higher degree of gold clustering, with the gold particles more frequently appearing in groups of three or more in both neurons and muscles (Figures 4C–4G, 4J, and 4K, Supporting Figure 2B). In contrast, there were very few incidences of clusters with three or more gold particles in the full-length receptor samples (Figures 4C, 4D, and 4J). In the Δ363–379 deletion strain, GFP-labeled receptor clusters were most frequently associated with intracellular membrane-bound compartments rather than clearly localized to the plasma membrane (Figures 4J and 4K). Based on their morphology and localization, these compartments may correspond to endosomal, vesicular, or other intracellular trafficking-associated membrane structures. As a control, immuno-EM was also performed on wild-type (WT) *C. elegans* (N2) animals that did not express GFP. In these samples, very few gold particles were detected overall (supporting Figure 2C), indicating high specificity of the antibody labeling for GFP. Although limited in its throughput (*n* = 2 per strain), immuno-EM provides compelling high-spatial resolution evidence that this region of the M3-4 loop of LGC-50 plays a crucial role in its intracellular localization.

### Truncation of residues 363–379 in LGC-50 does not interfere with aversive olfactory learning

It was previously reported that truncation of residues 363–379 in LGC-50 markedly increases the serotonin-induced current when the receptor is expressed heterologously in *Xenopus laevis* oocytes,^36^ confirming that the truncated receptor remains functional. Given that *lgc-50* (*tm3712*) null mutant animals display defects in aversive olfactory learning,^36^ we wondered whether the increased clustering observed for the truncated receptor might influence its function *in vivo* in a similar way.

**Figure 5:**
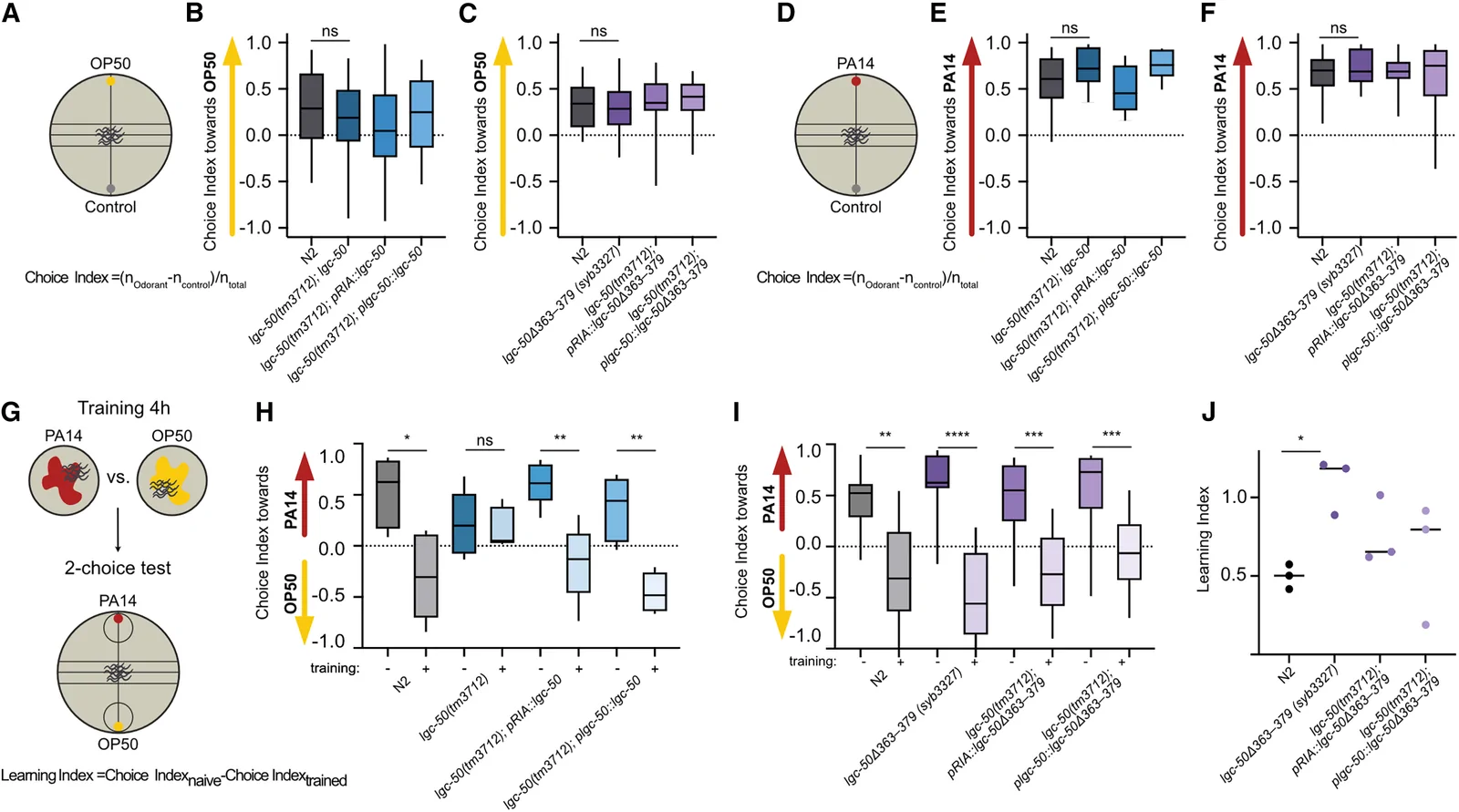
LGC-50Δ363–379 worms are capable of two-choice aversive olfactory learning (A) Schematic representation of experimental design. (B and C) Chemotaxis toward OP50 for N2, the null mutant for *lgc-50*: *lgc-50* (*tm3712*), the RIA-specific *lgc-50* rescue line: *lgc-50* (*tm3712*); *pglr-6::lgc-50::SL2mKate2*, the *lgc-50* rescue line under its own promotor: *lgc-50* (*tm3712*); *plgc-50::lgc-50::SL2mKate2*, the LGC-50 M3-M4 loop truncation mutant*: lgc-50Δ363–379* (*syb3327*), the RIA-specific *lgc-50* rescue line with a loop truncated version of LGC-50: *lgc-50* (*tm3712*); *pglr-6::lgc-50Δ363–379::SL2mKate2*, and the *lgc-50* rescue line under its own promoter with a loop-truncated version of LGC-50: *lgc-50* (*tm3712*)*; plgc-50::lgc-50Δ363–379::SL2mKate2*. *N* = 17–30 plates/genotype. (D) Schematic representation of experimental design. (E and F) Chemotaxis toward PA14. *N* = 14–25 plates/genotype. (G) Schematic representation of experimental design. (H and I) Calculated choice indices after two-choice aversive olfactory learning toward PA14. *N* = 5–15 plates per genotype and learning condition. (J) Learning index calculated from choice index after aversive olfactory learning. Each experiment was replicated over three days. ∗*p* < 0.05., ∗∗*p* < 0.01, ∗∗∗∗*p* < 0.0001; ns, no significant difference, calculated by Welch’s *t* test, data are shown as mean ± SEM.

To address this, we assessed the animals’ ability to perform chemotaxis (CTX) toward both their standard food source *Escherichia coli* OP50 and the pathogenic bacteria PA14 (Figure 5). As a reference, we included the *lgc-50* (*tm3712*) null strain, which has previously been shown to exhibit normal CTX toward OP50 but impaired aversive learning.^36^ Both the *lgc-50* null and the *lgc-50::Δ363–379* truncated worms showed normal navigation toward OP50 and PA14, indicating intact basal CTX (Figures 5A–5F). To confirm that the day-to-day reliability was high, all behavioral experiments were performed over three individual days (supporting Figures 3A–3H).

Next, we tested the Δ363–379 strain’s ability to perform aversive olfactory learning using the two-choice assay to measure olfactory preference to PA14 and OP50 after 4 h of pre-exposure to PA14 (Figures 5G–5J). Surprisingly, the *lgc-50::Δ363–379* worms performed significantly better than N2 controls, exhibiting roughly double the learning index score in this assay (Figure 5J). This suggests that the truncated receptor enhances the worms’ ability to remember the PA14 odor and associate it with negative experiences at this acute time point. As a control for assay conditions, we again included the *lgc-50* (*tm3712*) strain, which reproduced the expected learning-deficient phenotype, while expression of WT *lgc-50* in the RIA interneurons fully rescued the learning-deficient phenotype (Figure 5H).

### Training with pathogenic exposure increases fluorescent puncta of LGC-50 in WT worms but not in the Δ363–379 truncated receptor

**Figure 6:**
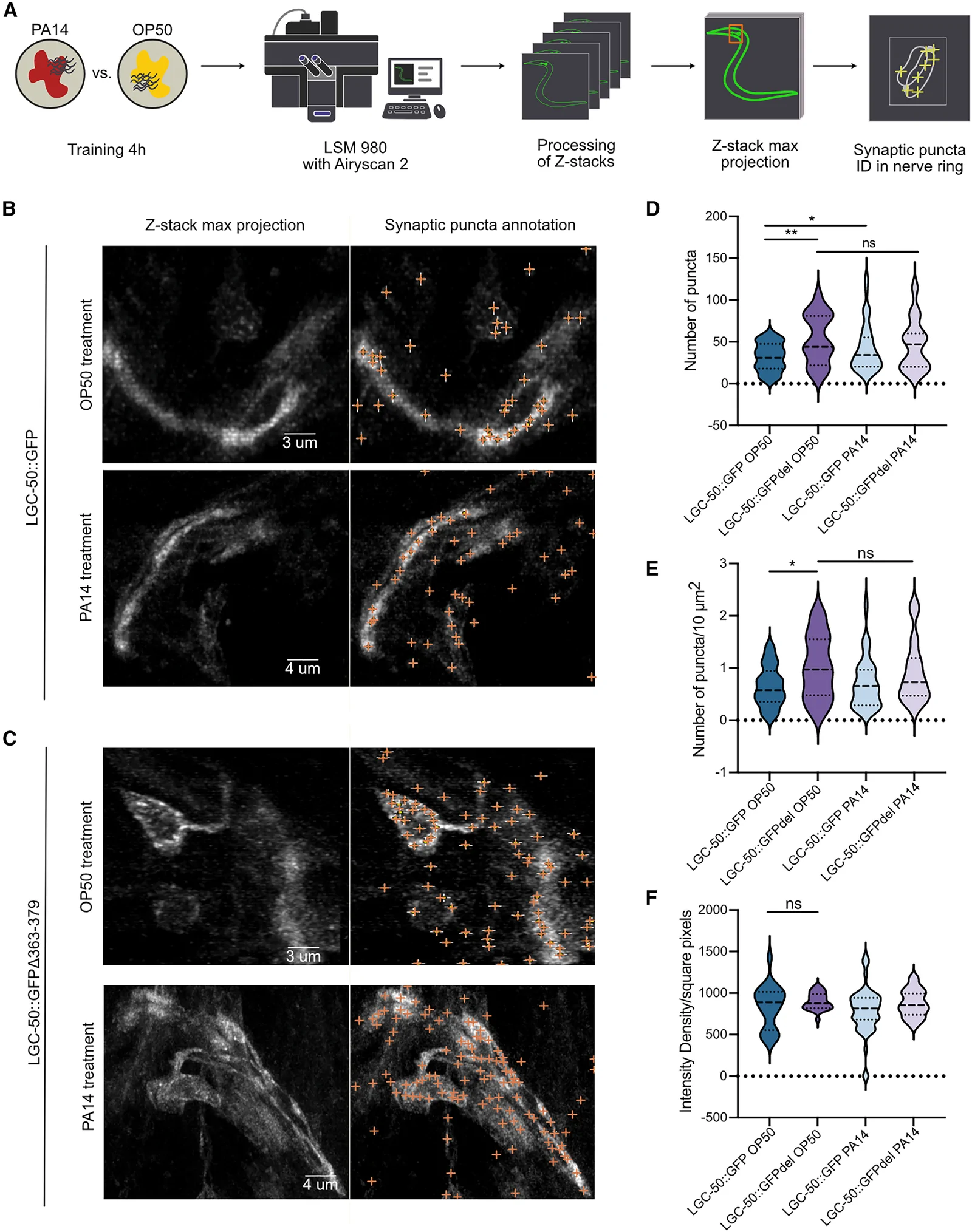
Exposure to PA14 for 4 h increases fluorescent puncta of *lgc-50::GFP* (*Ij120*) in the nerve ring but not for TM3-4 loop truncated *lgc-50* (*syb3377*) (A) Flowchart describing the work process for puncta quantification with PA14 exposure. (B) Representative images of LGC-50:GFP worms with and without PA14 exposure. (C) Representative images of LGC-50:GFPΔ363–379 worms with and without PA14 exposure. (D) Average total number of detected puncta/worm with and without PA14 exposure. (E) Average number of puncta normalized to area/worm with and without PA14 exposure. (F) Total fluorescence measured in the nerve ring. *N* = 29 worms LGC-50:GFP_OP50_, *N* = 19 worms LGC-50:GFP_PA14,_*N* = 51 LGC-50Δ363–379_OP50_, *N* = 30 worms LGC-50Δ363–379_PA14_, presented as a violin plot with median, 1^st^ and 3^rd^ quartile marked with dashed lines, ∗*p* < 0.05, ∗∗*p* < 0.01; ns, no significant difference, calculated using an unpaired *t* test.

To further investigate whether receptor trafficking is altered by PA14 exposure, we exposed animals to PA14 or OP50 for 4 h and immediately imaged the nerve ring using light microscopy (Figure 6A). Quantification of fluorescent puncta was performed using the same semi-automated image analysis pipeline as previously described (Figures 6B and 6C).

Consistent with our earlier findings, the Δ363–379 truncated strain exhibited a significantly higher number and density of fluorescent puncta compared to WT animals (Figures 6D and 6E). However, in contrast to WT worms, in which PA14 exposure induced a robust increase in puncta number relative to OP50 exposure, the Δ363–379 strain showed no detectable change in puncta count or density following training (Figures 6D and 6E). Importantly, total fluorescence intensity did not differ between the two strains nor in the same strain before and after PA14 exposure (Figure 6F), indicating that the observed differences reflect receptor redistribution rather than altered protein expression levels. Together, these results demonstrate that pathogen-induced learning triggers a redistribution of LGC-50 in WT neurons, whereas the Δ363–379 truncation abolishes this plastic response. This provides further evidence that the intracellular motif deleted in the truncated receptor might play a crucial role in activity-dependent receptor trafficking and synaptic plasticity.

### LGC-50Δ363–379 is unable to retrieve memory already after 1 h

**Figure 7:**
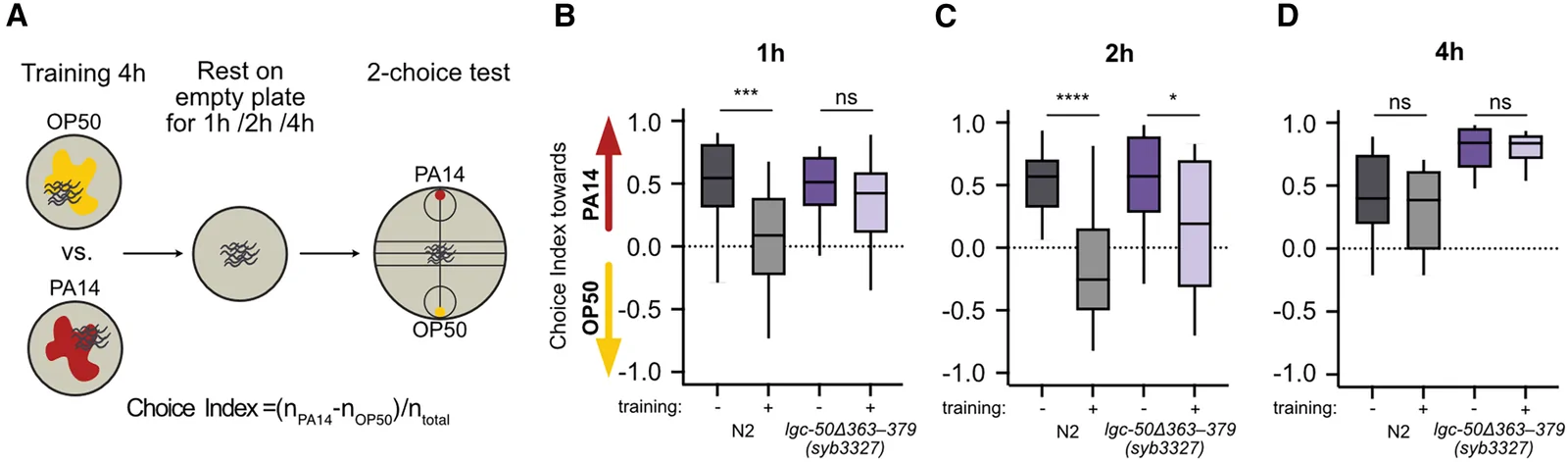
LGC-50Δ363–379 has impaired memory retrieval ability (A) Schematic representation of experimental design for two-choice assay with delayed memory test. (B–D) Choice indices after 1, 2, or 4 h for N2 and *lgc-50Δ363–379* (*syb3327*). *N* = 5–20 plates per genotype, learning condition and time point. Each experiment was replicated over three days. ∗*p* < 0.05, ∗∗∗*p* < 0.001, ∗∗∗∗*p* < 0.0001; ns, no significant difference, calculated by Welch’s *t* test, data are shown as mean ± SEM.

Given that our initial learning data suggested that the *lgc-50::Δ363–379* strain exhibited normal or even enhanced aversive learning, we next assessed its memory retention over time, as a measure of synaptic strength and memory stability^43^^,^^44^ (Figure 7A). Animals were pre-exposed to PA14 for 4 h, then allowed to recover on OP50 plates for 1, 2, and 4 h before being tested in the two-choice olfactory assay. Consistent with previous reports,^45^ N2 worms retained the learned aversion and successfully discriminated between the two odors for up to 2 h after training (Figures 7B–7D). In contrast, the *lgc-50::Δ363–379* worms displayed impaired maintenance of pathogen memory at the 1-h time point (Figure 7B), corresponding to 1 h of recovery between the end of the PA14 pre-exposure and the start of the two-choice testing, as well as reduced memory recall at the 2 h time point (Figure 7C), and showed complete loss of memory at the 4 h time point (Figure 7D).

This finding indicates that, while the Δ363–379 truncation does not impair the acquisition of aversive learning, it causes a rapid decay in memory retrieval, suggesting a defect in synaptic consolidation or maintenance of memory-related plasticity.^46^ One possible explanation is that the truncation disrupts learning-induced receptor trafficking between intracellular membrane compartments and synaptic membrane domains, thereby compromising the reinforcement of synaptic connections necessary for sustained memory retention. Our findings show how molecular changes in receptor localization can manifest as measurable alterations in behavior, offering direct evidence linking synaptic plasticity mechanisms to memory expression in a living organism.

### Interaction partners to LGC-50 include proteins of importance for synaptic strength and for the secretory pathway

**Figure 8:**
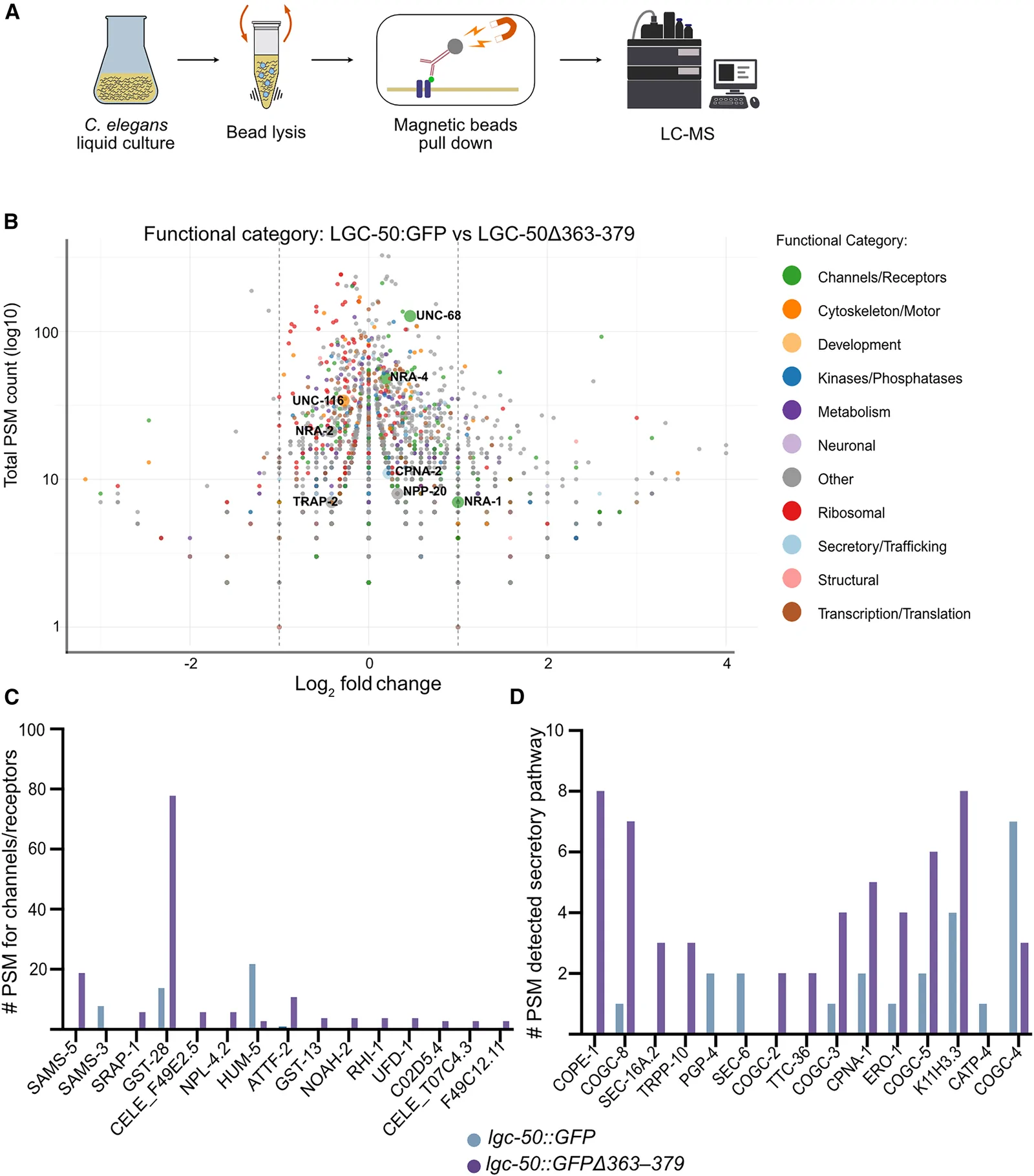
Immunoprecipitation with LC-MS analysis of *lgc-50::GFP* (*Ij120*) and *lgc-50::GFPΔ363–379* (*syb3377*) (A) Workflow for immunoprecipitation experiments in *C. elegans*. (B) Log_2_ fold change calculation of total PSM count. (C) Quantitative comparison of the proteins that differ the most between genotypes for the channel- or receptor-relevant proteins. Key proteins of biological interest with significant fold changes are highlighted in the volcano plot. (D) Quantitative comparison of the proteins that differ the most between genotypes for secretory-relevant proteins. Analysis was performed with pseudocount to handle zero values. Significance threshold was set to ± 1.0 log_2_ units (corresponds to ∼2-fold change). Due to single replicate design, traditional statistical significance testing was not performed.

The intracellular M3-4 loop of various LGICs has been repeatedly implicated in receptor localization, membrane trafficking, and protein complex assembly, both through interactions with other proteins^47^ and via distinct amino acid motifs within the loop itself.^23^^,^^48^ Based on our imaging and behavioral data, the 17-residue segment of the M3-4 loop in LGC-50 (Figure 1) appears to be critical for membrane receptor trafficking and the stabilization of synaptic connections with implications for memory retrieval. To identify potential binding partners to the LGC-50 interactome that could mediate these processes, we cultured large volumes of GFP-tagged *C. elegans* expressing either full-length or Δ363–379 truncated LGC-50 protein. Proteins were immunoprecipitated using anti-GFP magnetic beads and subsequently analyzed by liquid chromatography-mass spectrometry (LC-MS) (Figure 8A). Both strains yielded a comparable number of interacting proteins (supporting Figure 4), though differences were observed in the abundance and identity of specific binding partners (Figures 8B–8D). The total count of peptide spectrum matches (PSMs) was duplicate corrected and compared between the two genotypes. Several proteins of interest were found to be interacting with LGC-50 (highlighted in Figure 8B), such as proteins reported to be part of a multipass membrane protein machinery (NRA-2, nicalin) that is involved in the insertion and folding at the endoplasmic reticulum.^49^ Furthermore, UNC-116 was identified as a potential protein partner. UNC-116 has been shown to be involved in modulating synaptic strength through its involvement in rapid delivery, repositioning, and removal of receptors to the plasma membrane.^50^ We also identified the copine protein NRA-1, which has previously been reported to interact with LEV-1,^51^ a *C. elegans* nicotinic acetylcholine receptor. Copine proteins are known for being involved in directing proteins to plasma membranes upon Ca^2+^ binding,^52^ which could fit with an activity-dependent plasma membrane trafficking model for LGC-50. To build hypotheses on the interactome of LGC-50 and protein partners that could be of importance for regulating its membrane localization we specifically focused our analysis on proteins potentially involved in ion channel and receptor function, or proteins known to be part of the secretory pathway. We compared the differential interaction between the full-length protein and the loop deletion (Figures 8C and 8D) and found several interesting proteins in these two classes to be differently pulled down with either of the protein versions. Together, these data suggest that LGC-50 interacts with components of both the synaptic and secretory machinery, highlighting a potential molecular framework through which intracellular receptor partners coordinate trafficking, localization, and ultimately, memory-related plasticity.

## Discussion

Neural circuits continuously adapt their function in response to experience, a process known as synaptic plasticity, which underlies learning and memory formation. At the cellular level, plasticity often arises from activity-dependent redistribution of neurotransmitter receptors at the synapse. In this study, we identify a short intracellular motif within the M3-4 loop of the serotonin-gated ion channel LGC-50 as a critical determinant of receptor trafficking and memory retrieval in *C. elegans*. By combining electron and light microscopy, behavioral assays, and proteomic analysis, we show that deletion of residues 363–379 alters receptor localization, abolishes learning-induced redistribution, and leads to impaired memory retrieval without affecting initial learning. This rapid memory decay reflects a deficit in memory retention rather than acquisition and might be consistent with impaired receptor redistribution or stability at synaptic sites.

Our imaging data demonstrate that deletion of residues 363–379 in LGC-50 leads to increased receptor clustering and a pronounced accumulation of receptors in intracellular compartments, likely representing endosomal or pre-synaptic vesicular pools. Despite normal overall expression levels, these receptors fail to redistribute following pathogen exposure, in contrast to WT animals, which display a clear increase in receptor puncta after learning. However, our imaging approaches do not allow definitive assignment of these receptor populations to specific membrane compartments. While the biotinylation experiments in *Xenopus* oocytes support altered plasma membrane localization of the truncated receptor, the *in vivo* fluorescence and immuno-EM analyses suggest that a substantial fraction of LGC-50Δ363–379 is associated with intracellular membrane-bound compartments, potentially linked to receptor trafficking or vesicular sorting pathways. We therefore cannot conclude that the increased clustering observed *in vivo* exclusively reflects enhanced synaptic plasma membrane localization. The behavioral consequences of this mislocalization are notable as the truncated receptor supports normal or even enhanced learning but cannot sustain memory retrieval over time. Together, these findings indicate that this short intracellular motif is necessary for activity-dependent receptor trafficking and stabilization of memory-related synaptic changes.

Our data highlight the M3-4 intracellular loop as a critical regulatory region for receptor localization and trafficking. This loop is known to mediate protein-protein interactions, quality control of assembly, and trafficking signals across multiple ion channel.^53^^,^^54^ Within this region, specific short motifs often act as endoplasmic reticulum (ER) retention or export signals. Arginine-based retention motifs such as RxR or RKR have been shown to prevent ER exit for various channels, including K_ATP_ and GABA_B_ receptors, until masked by the correct subunit.^48^^,^^55^ The deleted segment in LGC-50 contains an RKR sequence, suggesting that this region may function as a conditional retention motif regulating ion channel receptor export from the ER. Removal of this motif could lead to premature or unregulated trafficking, resulting in accumulation of receptors in intracellular compartments. Additionally, the deleted segment includes serine and threonine residues, which could serve as phosphorylation sites influencing trafficking or receptor mobility. Similar regulatory mechanisms are well described for AMPA-type glutamate receptors, where phosphorylation of GluA1 by PKC or PKA promotes surface insertion during long-term potentiation.^56^^,^^57^ Whether analogous phosphorylation-dependent mechanisms exist for LGC-50 remains to be determined, but such regulation could explain the activity-dependent redistribution observed in WT animals.

The trafficking pattern we observe for LGC-50 resembles activity-dependent receptor dynamics seen in mammalian systems. In hippocampal neurons, AMPA receptors are inserted into the plasma membrane during learning through the conventional secretory pathway, and then move laterally to synapses.^58^^,^^59^ Distinct AMPA receptor subunits display different trafficking behaviors; for example, GluA1 rapidly exits the ER and reaches the plasma membrane,^60^ whereas GluA2 is more tightly regulated and often retained in intracellular puncta.^61^^,^^62^ These differential dynamics fine-tune synaptic strength and contribute to the persistence of memory traces. Analogously, LGC-50 undergoes learning-induced redistribution to synaptic regions in response to exposure to the pathogen *Pseudomonas aeruginosa* PA14. The failure of the Δ363–379 receptor to respond to this training suggests that the deleted motif is essential for coupling receptor localization to experience-dependent signaling. Thus, LGC-50 provides a tractable model to link molecular trafficking events to behavioral plasticity, allowing visualization of receptor dynamics *in vivo* at synaptic resolution. Behaviorally, the Δ363–379 truncation separates learning from memory retention. Worms expressing the truncated receptor can acquire an aversive association but rapidly lose it within an hour, indicating that while synaptic signaling may initially be intact, synaptic stabilization mechanisms are compromised. This parallels mechanisms observed in vertebrate systems, where maintenance of potentiated synapses depends on sustained receptor insertion, retention, and recycling at the postsynaptic membrane.^48^^,^^63^ The inability of LGC-50Δ363–379 to redistribute after learning might thus prevent reinforcement and the strengthening of synaptic connections, leading to a failure to reactivate the relevant neuronal circuits during memory retrieval.

In WT animals, exposure to PA14 resulted in a marked increase in LGC-50 puncta, suggesting that learning triggers a redistribution of the receptor into discrete intracellular or synaptic compartments. An important question is whether these puncta persist for as long as the learned aversive memory is maintained and they subsequently dissipate once the memory fades. Such a temporal correspondence would support the idea that the observed puncta reflect the dynamic flow of receptors between intracellular pools and synaptic membranes, thereby mirroring the molecular consolidation and decay of memory. The intracellular localization of these puncta may also indicate association with endosomal or vesicular compartments, consistent with receptor trafficking through recycling or sorting pathways rather than stable accumulation at the plasma membrane. If so, the increased puncta observed after PA14 exposure could represent a transient accumulation of receptors in trafficking intermediates that accompany activity-dependent redistribution to the synapse. In contrast, the absence of such regulation in the Δ363–379 mutant might indicate that the truncated receptor is trapped within these compartments and unable to complete the trafficking cycle required for synaptic insertion. This scenario would explain why receptor clustering is elevated but static in the mutant and support a model in which the 17-amino acid intracellular motif enables the dynamic receptor cycling necessary for maintaining memory-related synaptic plasticity.

Our proteomic analysis identified several proteins that differentially associate with full-length versus truncated LGC-50. Among these were components of the ER membrane complex (EMC), such as NRA-2/nicalin, implicated in the insertion and folding of multi-pass membrane proteins,^49^ and UNC-116, a kinesin motor protein involved in vesicular transport and synaptic receptor delivery.^50^ These interactions suggest that LGC-50 trafficking is coordinated with cellular machinery that regulates protein folding, transport, and synaptic positioning. Disruption of these interactions could underlie the altered localization observed for the truncated receptor.

Although the precise mechanism by which LGC-50 enters the secretory pathway remains unresolved, our findings indicate that regulated receptor trafficking is required for maintaining but not acquiring learned behavior. The redistribution of LGC-50 after pathogenic exposure likely reflects a dynamic trafficking event linked to learning. The intracellular puncta observed may represent transient endosomal or vesicular pools involved in receptor recycling rather than stable synaptic membrane insertion. We propose that, after learning, LGC-50 cycles through these compartments toward synaptic sites, whereas in the Δ363–379 mutant, receptors become trapped in these intermediates, preventing the trafficking needed for memory maintenance.

By directly linking receptor trafficking to behavioral plasticity, this study reveals how molecular events at the level of individual receptors translate into network-level memory stability. More broadly, our findings provide a mechanistic framework for understanding how activity-dependent trafficking of neurotransmitter receptors underpins the persistence and retrieval of memory, a principle likely conserved across nervous systems from nematodes to mammals.

### Limitations of the study

Although our combined behavioral, fluorescence imaging, immuno-EM, and biochemical analyses support a role for the intracellular M3-4 loop of LGC-50 in receptor trafficking and memory retrieval, several limitations should be considered. First, our imaging approaches cannot definitively resolve the dynamic movement of LGC-50 between specific membrane compartments *in vivo* and therefore do not distinguish whether the observed receptor clusters represent endosomal, vesicular, or synaptic membrane-associated pools. Second, while surface biotinylation in *Xenopus* oocytes demonstrates that deletion of residues 363–379 increases plasma membrane localization in a heterologous system, the subcellular distribution of the mutant receptor in *C. elegans* likely reflects a more complex trafficking equilibrium. Finally, although our proteomic analysis identified several candidate trafficking partners, these interactions remain correlative and will require future biochemical and genetic validation to establish their functional roles in LGC-50 localization and memory-related plasticity.

## Resource availability

### Lead contact

Further information and requests for *C. elegans* strains and plasmids should be directed to and will be fulfilled by the lead contact, Julia Morud (julia.morud@gu.se).

### Materials availability

Materials generated in this study, including strains, plasmids, and clones, are freely available from the lead contact upon reasonable request.

### Data and code availability

• All experimental data, including the results of imaging, behavioral and biochemical experiments, will be shared by the lead contact upon request.
• This paper does not report original code.
• Any additional information required to reanalyze the data reported in this paper is available from the lead contact upon request.

## Acknowledgments

We gratefully acknowledge Lara Prüßner for help with cloning design and help with generating and maintaining strains and other past and present members of the Morud Lekholm lab for helpful discussions. We would also like to acknowledge Iris Hardege for critical discussions and valuable comments during manuscript writing. We would like to acknowledge the Center for Cellular Imaging at the University of Gothenburg, Sweden, and the National Microscopy Infrastructure (NMI), for providing imaging facilities. We would also like to thank the Proteomics Core Facility at the University of Gothenburg, Sweden, for assisting with mass spectrometry analysis. This work was supported by grants from the 10.13039/501100004359Swedish Research Council (2022–03951), Swedish Foundation for Strategic Research (FFL21-0166), Tore Nilssons stiftelse, Carl Tryggers Stiftelse (22:1966), Åke Wibergs stiftelse (M22–0215 and M21-0229), 10.13039/501100006285Magnus Bergvalls Stiftelse to J.M., the 10.13039/501100004359Swedish Research Council (2019–04004) and Cancer Foundation (21 1865 Pj01H) to J.L.H. and Lundgrenska stiftelserna to L.C.

## Author contributions

Conceptualization, J.M., L.C., and J.L.H.; data curation, L.C., D.Z., and A.C.; methodology, J.M., J.L.H., L.C., D.P., A.C., and A.K.; investigation, L.C., E.A., D.Z., A.C., and A.K.; formal analysis, L.C. and J.M.; visualization, L.C., J.M., D.Z., and A.C.; funding acquisition, J.M. and J.L.H.; project administration, J.M.; supervision, J.M.; validation, J.M. and J.L.H.; resources, J.M. and J.L.H.; writing – original draft, J.M.; writing – review and editing, J.M., L.C., D.Z., E.A., A.C., A.K., and J.L.H.

## Declaration of interests

The authors declare no competing interests.

## Declaration of generative AI and AI-assisted technologies in the writing process

During the preparation of this manuscript the authors used ChatGPT in order to improve text quality. After using this tool, the authors reviewed and edited the content as needed and take full responsibility for the content of the published article.

## STAR★Methods

### Key resources table

**Table:** 

REAGENT or RESOURCE	SOURCE	IDENTIFIER
**Antibodies**		

10-nm gold-conjugated goat anti-rabbit antibody	Aurion	Cat# 810.011; Lot# GAR-40530/1
GFP-Trap magnetic beads	Chromotek	Cat# gtma; Lot# 209072-02; RRID: AB_2631358
Anti-HA mouse antibody	Invitrogen	Cat# 32–6700; Lot# ZC374240; RRID: AB_2533092
HRP-conjugated goat anti-mouse antibody	Invitrogen	Cat# 31432; Lot# 096018I; RRID: AB_228302
HRP-conjugated polyclonal anti-β-actin antibody	Invitrogen	Cat# MA515739HRP; Lot# 3217311A; RRID: AB_10979409

**Bacterial and virus strains**		

*Escherichia coli* OP50	–	–
*Escherichia coli* 10-beta, competent cells	New England Biolabs	Cat# C3019I
*Pseudomonas aeruginosa* PA14	CCUG, University of Gothenburg	EUCPA

**Biological samples**		

*Xenopus laevis* defolliculated oocytes	Ecocyte Bioscience	N/A

**Chemicals, peptides, and recombinant proteins**		

Hexadecene	Merck	Cat# 8.22064; Lot# S6852764 412
Uranyl Acetate	Electron Microscopy Sciences	Cat# 22400;
Lowicryl HM20 resin	Polyscience	Cat# 15924A-225
Agar 100 resin	Agar Scientific	Cat# AGR1140B
Formvar	TAAB Laboratories Equipment	Cat# F005; Lot# 21870
Fish skin gelatin	Merck	Cat# G7765
Glutaraldehyde	Thermo Fisher Scientific	Cat# 11423488
Sodium azide	Thermo Fisher Scientific	Cat# 190381000; Lot# A0455064
Pepstatin A	Melford	Cat# P30100; Lot# D23869
Benzamidine hydrochloride	Sigma-Aldrich	Cat# B-6506; Lot# 60K1448
Phenylmethylsulfonyl fluoride (PMSF)	Roche	Cat# 11359061001; Lot# 54561620
Leupeptin	Sigma-Aldrich	Cat# EI8
NotI-HF restriction enzyme	New England Biolabs	Cat# R3189S; Lot# 10116600
EZ-Link Sulfo-NHS-Biotin	Thermo Fisher Scientific	Cat# A39256
Triton X-100	VWR	Cat# T6559-1
HiTrap Streptavidin HP	Cytiva	Cat# 17511201; Lot# 10233977
Laemmli sample buffer	Bio-Rad	Cat# 1610747
Tween 20	Thermo Fisher Scientific	Cat# 85114; Lot# ZI402047

**Experimental models: Organisms/strains**		

*C. elegans* N2	CGC	N/A
*C. elegans* lgc-50:GFP (insertion after codon G360)	Morud et al.^36^ Current Biology, 2021	Allele Ij120
*C. elegans* lgc-50:Δ[363–379]	SunyBiotech	Allele syb3327
*C. elegans* lgc-50:GFP::Δ[363–379]	SunyBiotech	Allele syb3377

**Oligonucleotides**		

Primer: Insertion of HA-tag in full-length lgc-50, fwd GCCCGACTACGCTGGAGGTGTTGGAGGCCTT	Eurofins Genomics	N/A
Primer: Insertion of HA-tag in lgc-50 Δ[363–379], fwd GCCCGACTACGCTGGAGGTGCTGCTACAGATTCG	Eurofins Genomics	N/A
Primer: Insertion of HA-tag in lgc-50, universal rev ACGTCGTAGGGGTAACCACCTCCTTCATCGGCTT	Eurofins Genomics	N/A
Primer: Amplification of lgc-50 cDNA for GFP assembly, fwd AAAGCAGGCTTAATGCGATTTCTTCTTGTTCTTCA	Eurofins Genomics	N/A
Primer: Amplification of lgc-50 cDNA for GFP assembly, rev AGAAAGCTGGGTTTTACATGGGACGATCCATTTTC	Eurofins Genomics	N/A
Primer: Amplification of GFP for lgc-50 assembly, fwd ATGAGTAAAGGAGAAGAATTGTTC	Eurofins Genomics	N/A
Primer: Amplification of GFP for lgc-50 assembly, rev GCCTCCACTACCGC	Eurofins Genomics	N/A

**Recombinant DNA**		

pDESTR4R3II vector	Schafer Lab, UK	–
KSM vector	Schafer Lab, UK	–
KSM lgc-50:HA-tag after codon G360	This paper	–
KSM lgc-50:Δ[363–379]:HA-tag after codon G360	This paper	–

**Software and algorithms**		

IMOD image analysis software	Kremer et al.^64^	https://bio3d.colorado.edu/imod/
DBSCAN algorithm	Ester et al.^65^	–
ZEN imaging and processing software	Zeiss	N/A
ImageJ/Fiji image analysis software	Schneider et al.^66^	https://imagej.nih.gov/ij/
ImageJ macro	Alyona^39^; Zou^40^	–
Prism10	GraphPad Software	https://www.graphpad.com/features
R (v 4.0 or later)	R Foundation	https://www.r-project.org/
R package dplyr	N/A	https://github.com/tidyverse/dplyr
R package ggplot2	N/A	https://github.com/tidyverse/ggplot2
R package ggrepel	N/A	https://github.com/slowkow/ggrepel
R package readxl	N/A	https://github.com/tidyverse/readxl
R package writexl	N/A	https://docs.ropensci.org/writexl/
AlphaFold3	Jumper et al.^37^; Abramson et al.^38^	https://alphafoldserver.com/

**Other**		

Type A specimen carrier for high-pressure freezing	Wohlwend GmbH	Cat# 241
Type B specimen carrier for high-pressure freezing	Wohlwend GmbH	Cat# 242
PELCO Slot Grids, Copper, 1 × 2 mm, 3 mm Ø	Ted Pella	Cat# 1 GC12H
Q5 High-Fidelity Polymerase 2X Master Mix	New England Biolabs	Cat# M0492S; Lot# 10072059
Phusion Green Hot Start II High-Fidelity PCR Master Mix	Thermo Fisher Scientific	Cat# F-566S; Lot# 3399288
2X Phire Green Hot Start II PCR Master Mix	Thermo Fisher Scientific	Cat# F-126; Lot# 3140363
KLD Enzyme Mix	New England Biolabs	Cat# M0554S; Lot# 10283573
KLD Reaction Buffer	New England Biolabs	Cat# B0554A; Lot# 10249180
NEBuilder HiFi DNA Assembly Master Mix	New England Biolabs	Cat# M5520AA; Lot# 10238676
In-Fusion Snap Assembly Master Mix	Takara Bio	Cat# 638947
PureLink™ Quick Plasmid Miniprep Kit	Invitrogen	Cat# K210011; Lot# 2954291
Monarch® PCR & DNA Cleanup Kit	New England Biolabs	Cat# T1030L; Lot# 10208654
mMESSAGE mMACHINE™ T3 Transcription Kit	Invitrogen	Cat# AM1348; Lot# 3411626
RNA Clean & Concentrator-5 Kit	Zymo Research	Cat# R1013; Lot# 259635
Trans-Blot Turbo Buffer	Bio-Rad	Cat# 10026939; Lot# 64703947
TransBlot Turbo Mini size PVDF membrane	Bio-Rad	N/A
Restore™ PLUS Western Blot Stripping Buffer	Thermo Fisher Scientific	Cat# 46430; Lot# ZH396265

### Experimental model and study participant details

#### Animals

This study uses the nematode *Caenorhabditis elegans* as the experimental model. Unless indicated otherwise, hermaphrodite *C. elegans* worms were cultured at 20 °C on nematode growth medium (NGM), plates were seeded with *Escherichia coli* OP50. Transgenic rescue strains were produced by microinjecting plasmid DNA into the gonads of young adult hermaphrodites, and progeny carrying stable extrachromosomal arrays were subsequently selected. Offspring with stable array transmission were selected for isolated strains. The following alleles were generated by SunyBiotech (Fuzhou, China) using CRISPR/Cas9-based genome editing: lgc-50:Δ363–379(syb3327) and lgc-50:GFP::Δ363–379(syb3377). CRISPR generated lines were backcrossed to our laboratory stock of wild-type worms (N2) four generations before being used. All behavioral experiments were performed in day 1 adult hermaphrodite worms.

#### Bacterial strain

The *Escherichia coli* strain OP50 was grown in either 2XYT or Luria-Bertani (LB) broth at 37°C and used for seeding LB plates as a food source for the worms.

### Method details

#### Molecular biology

*C. elegans* gDNA, cDNA or promotor regions were amplified out of N2 DNA libraries using reverse transcription PCR with either Q5 polymerase (NEB, MA, USA) or Phusion and Phire polymerases (ThermoFisher Scientific). Gene fragments were assembled into the pDESTR4R3II vector (for expression in worms) and into the KSM vector (for expression in *Xenopus* oocytes) using the HiFi assembly protocol (NEB, MA, USA) or In-Fusion Snap assembly protocol (Takara, CA, USA). Unless specified, the promotor sequences used were approximately 2 kb upstream of the start codon.

Constructs used for surface biotinylation on *Xenopus* oocytes were produced via PCR insertion mutagenesis on KSM vector templates. An HA-tag was inserted in frame directly after codon G360 by amplifying the vector with primers with overhangs coding for the tag. Resulting PCR products were treated with the KLD Enzyme Mix (NEB, MA, USA) before transformation into competent *E. coli* 10-beta cells (NEB, MA, USA).

#### Electron microscopy

##### Sample preparation for Electron Microscopy (EM)

Day-1 adult worms were grown to high densities on NGM plates that had been generously seeded. The 0.1 mm cavity of the type A specimen carrier (Wohlwend GmbH, Switzerland) was filled with *C. elegans* and OP50 acting as a cryoprotectant^67^ using a worm pick. A Type B freezing carrier (Wohlwend GmbH, Germany) was dipped in hexadecene and placed flat side down on top of the carrier. The carriers were then frozen in a high-pressure freezer Compact 3 (Wohlwend GmbH, Switzerland) and transferred directly to the automatic freeze substitution (AFS) unit, (Leica, Germany). The samples were substituted with 2% uranyl acetate (Electron Microscopy Sciences, USA) dissolved in 10% methanol and 90% acetone,^68^^,^^69^ but with the adaptation that the uranyl acetate was left on the sample overnight at −90°C. The samples were then washed in acetone before stepwise infiltration at −50 °C with 20%, 40%, 50%, 80% and 100% freshly prepared Lowicryl HM20 resin (Polyscience, USA) diluted in acetone, 2 h per step. Infiltration continued overnight with 100% Lowicryl HM20 resin. UV polymerisation was initiated at −90 °C and continued during a controlled temperature rise to room temperature. Polymerisation proceeded for five days at room temperature. To reorient the sample, the top part of the sample cylinder containing the worms was cut off with a saw, and the edges were trimmed. The sample was then placed in a flat embedding mold in the desired orientation and re-embedded in Agar 100 resin (Agar Scientific, UK). The samples were cut into 70 nm ultra-thin sections using a diamond knife (Diatome, Switzerland) fitted to an Ultracut E microtome (Leica, Germany) and placed on a copper slot grid (PELCO, 1 × 2 mm, 3.0 mm O.D.) (Ted Pella, USA) coated with 1% formvar (TAAB, UK) in chloroform.

#### Immuno-EM

Thin sections were post-fixed in 1% paraformaldehyde in phosphate buffered saline (PBS) for 10 min, then washed 3 × 1 min in PBS. The grids were then blocked for 1 h in 0.1% fish skin gelatine (Merck, Germany) and 0.8% bovine serum albumin (BSA) in PBS. Immunolabeling was carried out by applying a 1:10 dilution in blocking solution of a rabbit anti-GFP antibody (Abcam, UK) overnight at 4 °C, followed by a three 20-min washes in PBS. Then a 1-h incubation at room temperature with a 1:20 dilution of a 10 nm gold-conjugated goat anti-rabbit secondary antibody (Aurion, Netherlands) was done. After another three 20-min washes in PBS, the sections were postfixed with 2.5% glutaraldehyde (Thermo Fisher Scientific, USA) for 1 h. Following 3 x 1-min washes in ddH_2_O, the grids were imaged.

#### EM image acquisition

Samples were imaged on a Tecnai T12 transmission electron microscope (Thermo Fisher Scientific, USA) equipped with a Ceta 16M CMOS camera (Thermo Fisher Scientific, USA).

#### Light microscopy

The worms were immobilised on 2% agarose pads using a solution of 75 mM sodium azide (Thermo Fisher Scientific, USA) in M9 buffer. Imaging was performed using a Zeiss LSM 980 with an Airyscan 2 detector and a 40× water-immersion objective (Zeiss, Germany). For trained samples, worms were exposed to *Pseudomonas aeruginosa* PA14 or OP50 training plates for 4 h under the same conditions as in the behavioral assay before imaging.

#### Chemotaxis

The preparation of plates and worm preparation and handling for the CTX assay was done as previously described,^70^ with some modifications. Fresh overnight liquid cultures of PA14 and OP50 were prepared from single colonies the day before the experiment. The following morning, the cultures were diluted and regrown until they reached an OD600 of 1. For the CTX assays, 25 μL of the PA14 or OP50 cultures (OD600 = 1) were spotted onto the assay plates and paired with 25 μL of lysogeny broth (LB) medium as a control. The plates were used in the assay within max. 2 h after preparation. The day-1 adult hermaphrodites (∼50) were washed from the plate with M9 and transferred to the assay plates. After running the assay for 1h 15 μL of sodium azide was dropped on the bacteria spots and the plates were stored at 4°C.

#### Aversive olfactory training and learning

The aversive training with the pathogenic bacterium PA14 was performed as previously described,^33^^,^^36^^,^^71^ with some modifications. To prepare the training plates, 100 μL of PA14 or OP50 culture was spotted onto NGM agar and incubated at 25 °C for two days. For the day of the experiment fresh overnight liquid cultures of PA14 and OP50 were prepared from single colonies. In the morning, the cultures were diluted and regrown until they reached OD_600_ of 1. For the learning assays, 25 μL of the PA14 and OP50 cultures (OD_600_ = 1) were spotted onto opposite sides of the assay plates. The assay plates were incubated at room temperature during the training period or during worm washing and were always used within max. 2 h after preparation. Worm handling, washing and transfer to the assay plates followed previously described procedures,^70^ except that the assays were performed on NGM plates rather than chemotaxis (CTX) plates. On the day of the experiment ∼70 days-1 adult hermaphrodites were transferred to PA14 or OP50 plates and exposed for 4 h at room temperature before testing. The worms were washed from the training plates with M9 and transferred to the assay plates. After 1h 15 μL of sodium azide was dropped on the bacteria spots and the plates were stored at 4°C. For quantification, only worms within a 25 mm radius around each bacterial spot were included. For the time series memory retrieval assay, worms were washed from the training or control plates and placed on an empty plate with a small amount of OP50 during the incubation time before the test, after which they were washed off the plate and tested in the two-choice assay as described above.

#### Immunoprecipitation from *C. elegans*

Large-scale worm cultures were prepared in liquid media, as previously described.^72^ Synchronised worm populations were grown in S medium supplemented with high concentrations of OP50 under vigorous shaking for four to five days, until the majority of the cultured worms were adults. The worm cultures were then chilled on ice, pelleted by centrifugation, washed with M9 (3 g KH_2_PO_4_, 6 g Na_2_HPO_4_, 5 g NaCl, 1 mL 1 M MgSO_4_, H_2_O to 1L). Pellets were resuspended in lysis buffer (10 mM Tris-HCl, 150 mM NaCl, 5 mM EDTA, 0.5% NP40) supplemented with a homemade protease inhibitor cocktail (200 μM Pepstatin A, 200 mM Benzamidine hydrochloride, 100 mM PMSF, 60 μM Leupeptin, all in 95% ethanol) and 0.5 mm glass beads (Biospec, USA) were added. Lysis was performed using a FastPrep-24 5G (MPbio, USA) with 5.5 m/s for 120 s with 180 s break repeated for 3 times at 4°C.

For co-immunoprecipitation, the worm lysates were incubated with GFP-Trap magnetic beads (Chromotek, Germany) in end-to-end rotation at 4 °C for 1 h. The beads were then separated from the total lysate and washed in lysis buffer and eluted from the beads using SDS sample buffer (Biorad, USA). The eluates were sent for analysis at the Proteomics Core Facility at the Sahlgrenska Academy, University of Gothenburg, Sweden, for liquid chromatography mass spectrometry (LC-MS) analysis.

#### cRNA production and protein expression in Xenopus oocytes

KSM vectors carrying LGC-50 constructs were purified with the PureLink Quick Plasmid Miniprep Kit (Invitrogen). Then, they were linearised with NotI-HF restriction enzyme (New England Biolabs) and linear DNA was cleaned with the Monarch PCR & DNA Cleanup Kit (New England Biolabs). cRNA of LGC-50 constructs was produced from linearised KSM vectors using the mMESSAGE mMACHINE T3 Transcription Kit (Invitrogen) and the RNA was finally cleaned using the RNA Clean & Concentrator-5 Kit (Zymo Research).

Visually inspected oocytes (≥1 mm) were purchased from Ecocyte Bioscience. Upon arrival, oocytes were immediately washed in ND96 buffer (5 mM HEPES, 96 mM NaCl, 1 mM MgCl_2_, 1 mM CaCl_2_, 2 mM KCl) and transferred onto 96-well injection plates. Oocytes were injected with 50 nL of 500 ng/μL cRNA of either LGC-50 construct (full-length or truncated) or with water as a negative control and incubated for 72 h at 15 °C.

#### Biotinylation assay

After incubation, 20 oocytes per condition were washed in ice-cold phosphate-buffered saline (PBS). For each condition, half of the cells were resuspended in 200 μL PBS and the other half in 200 μL PBS supplemented with 2.5 mM EZ-Link Sulfo-NHS-Biotin. After an overnight incubation at 4 °C, three wash steps with 0.1 M glycine were performed to remove excess biotin and quench the reaction. Cells were then resuspended in PBS supplemented with 80 mM sucrose, 1 mM EDTA and a homemade protease inhibitor cocktail (200 μM Pepstatin A, 200 mM Benzamidine hydrochloride, 100 mM PMSF, 60 μM Leupeptin, all in 95% ethanol). Cells were lysed using a Dounce homogenizer (30 strokes), and the yolk was pelleted at 200 × g for 4 min. Triton X-100 was added to a final concentration of 1%, and samples were incubated for 1 h at 4 °C on a tilting table. To purify biotinylated proteins, 20 μL of settled streptavidin resin was added to each tube and incubated with the samples for 1 h at 4 °C. The binding step was followed by three washes with PBS and elution, which was performed by boiling the membrane in Laemmli buffer (supplemented with 50 mM DTT). Aliquots of the sample, application flow-through and elution were taken for SDS–PAGE analysis. The biotinylation experiments were performed over three individual days, with new batches of oocytes for each experiment.

#### Western blot analysis

Blotting was performed using a Trans-Blot Turbo transfer system. The PVDF membrane was first blocked in TBS-Tween (Tween 20 from Thermo Fisher Scientific) with 5% fat-free milk and incubated with primary anti-HA antibody (1:1000) and secondary antibody conjugated with horseradish peroxidase (1:1000). For β-actin detection, the PVDF membrane was stripped using Restore PLUS Western Blot Stripping Buffer for 10 min at room temperature, then blocked in TBST with 3% BSA and incubated with HRP-conjugated polyclonal anti-β-actin antibody (1:5000).

### Quantification and statistical analysis

All values in the text and figure legends are shown as mean ± standard error of the mean (SEM). The difference in the figures is represented using asterisks according to: ∗*p* < 0.05. ∗∗*p* < 0.01, ∗∗∗*p* < 0.001, ∗∗∗∗*p* < 0.0001, ns = no significant difference. The representation of N is indicated in the figure legend at each figure.

#### LGC-50 surface expression

Protein quantification was performed using Fiji.^73^ From the Western blot, the amount of LGC-50 was calculated according to the following equation: Amount of LGC-50 = Integrated Density − (Area of ROI × Mean Background) where ROI represents the region of interest encompassing the protein band. Each LGC-50 value was then normalised to the total protein content loaded in the corresponding gel lane. Total protein was quantified by selecting the entire lane as the ROI while excluding artifacts, including detergent-induced smearing and streptavidin signals originating from the streptavidin resin used during protein purification.

The percentage of biotinylated LGC-50 present at the cell surface was calculated using the following equation: % Surface LGC-50 = [(LGC-50_biotin – LGC-50_non-biotin)/(LGC-50_biotin – LGC-50_non-biotin + LGC-50_flow-through)] × 100 where LGC-50_biotin is the amount of LGC-50 detected in the biotin-treated sample, LGC-50_non-biotin is the amount detected in the non-biotinylated control sample, and LGC-50_flow-through is the amount of LGC-50 detected in the application flow-through from the biotin-treated sample. Surface expression measurements were performed in three independent biological replicates (*n* = 3). For each experimental condition, samples were prepared from 10 oocytes.

#### EM image analysis

Tissue areas were manually traced in IMOD^64^ and total contour areas were used for normalisation. Gold particles were annotated manually in IMOD and assigned to different compartments. Gold particle clusters were identified using the DBSCAN algorithm^65^ with a physical distance threshold of 70 nm and a minimum cluster size of three particles. Each cluster was classified according to the dominant tissue type of its constituent particles. Gold particle density and cluster density were calculated as counts per μm^2^ of tissue.

#### Light microscopy

The raw images were processed using ZEN software (Zeiss, Germany) before maximum intensity z-projections were generated in Fiji/ImageJ^66^ for analysis. Semi-automated puncta quantification was carried out using an ImageJ macro for single channel analysis.^39^^,^^40^ For each animal, a single region of interest (ROI) covering the nerve ring and associated cell bodies was selected. The macro quantified the total number of puncta and puncta density (puncta per 10 μm^2^). The settings used for the analysis were: ROI size: 20 × 10 μm with the prominence value for “find maxima” set to 35, and minimum accepted puncta size to 0.3. To ensure consistency, images were cropped as necessary to match the magnification across the dataset. The data was exported to Prism10 (GraphPad Software, MA, USA) and statistically compared using a paired *t* test.

#### Behavioral analysis

Chemotaxis and learning index was calculated as described before using GraphPad Prism 11.^36^ At the end of each assay, worm distributions were counted, and a choice index (CI = (n_test_ -n_control_)/n_total_) was calculated. The learning indices were defined as the difference between the choice index of untrained and trained animals.

#### Immunoprecipitation analysis

Raw data files contained quantitative metrics for each identified protein, including peptide counts, Peptide Spectrum Matches (PSMs), and unique peptide counts. Gene names were extracted from UniProt’s protein description using pattern matching for the “GN = ” identifier. To prevent artificial inflation of fold-change values, duplicate protein entries were identified based on matching gene names and subsequently aggregated.

Because the experiment was performed with a single replicate per condition, traditional significance testing was not applied. Instead, protein abundance differences were assessed using fold-change analysis, calculated as log_2_FC = log_2_((PSM_LGC50Δ363-379}+ 1)/(PSM_LGC50 + 1)), where pseudocounts (+1) were added to avoid division by zero. Proteins were categorised into 11 functional groups based on keyword matching. Keyword searches incorporated both domain-specific and functional terms such as “kinase,” “channel,” “receptor,” “transporter,” “ribosomal,” “cytoskeleton,” and “metabolism.” All visualisations were generated in R using the ggplot2 package. Volcano plots were created with log_2_ fold change on the x axis and total PSM count (log_10_ scale) on the y axis, with proteins color-coded by functional category. Protein abundance comparisons between conditions were visualised using log_10_-transformed PSM counts, and correlation analyses were performed using both Pearson and Spearman methods to assess data consistency. All analyses were conducted in R (version 4.0 or later) using dplyr for data manipulation, ggplot2 and ggrepel for visualisation, and readxl and writexl for Excel file handling.
