# Supplementary material for: Intracellular regulation of a serotonin-gated ion channel links receptor trafficking to memory
Source: iScience. 2026 Aug 25;29(9):117309. doi: 10.1016/j.isci.2026.117309 (PMC13542505; doi:10.1016/j.isci.2026.117309)
Supplement: Supplementary file 1 [file mmc1.pdf]

iScience, Volume *29*

Supplemental information

Intracellular regulation of a serotonin-gated ion channel links receptor trafficking to memory

Leona Cesar, Davide Zabeo, Andrea Cellini, Emelie Aspholm, Alexander Kolsrud, Dimitra Panagaki, Johanna Louise Höög, and Julia Morud

Supporting Figures and Tables

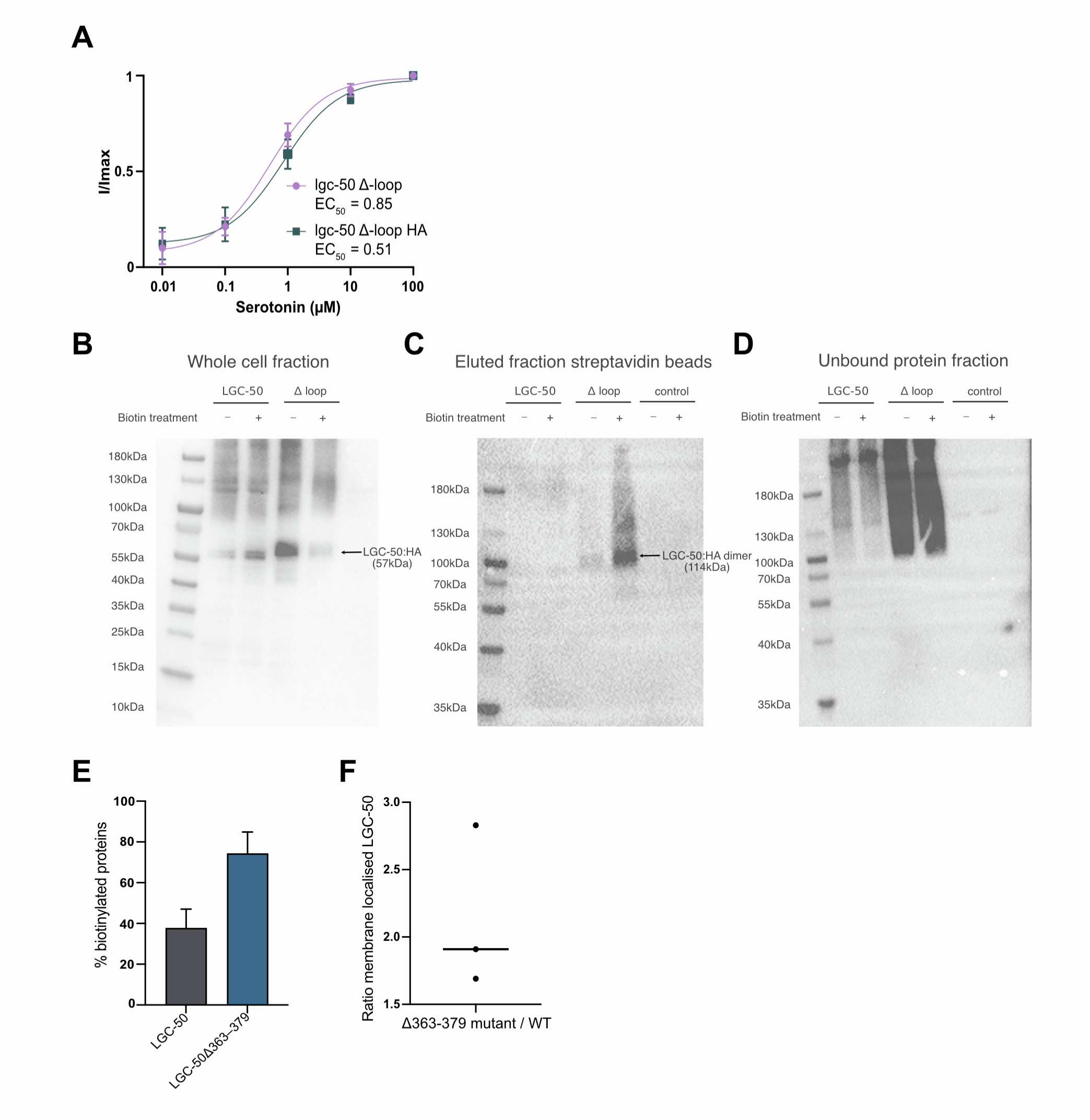

Supporting Figure 1. **Control experiments for tag insertion and biotinylation assay.** A. Tagging LGC-50Δ363-379 with an HA-tag in the M3M4 loop did not interfere with ion channel function. B. Total whole cell LGC-50::HA detected with anti-HA antibody shows LGC-50 monomeric protein at 57 kDa. C. Western blot using anti-HA antibodies after streptavidin pull-down eluted fraction shows LGC-50 dimer protein at approximately 114 kDa. D. Western blot using anti-HA antibodies after streptavidin pull-down unbound fraction. All Western blot was done using PageRuler Prestained Protein Ladder (ThermoFisher Scientific). E. Percentage of total LGC-50 that surface expressed represented by biotinylation. F. Ratio of LGC-50 localised to the plasma membrane in LGC-50::GFPΔ363-379 compared to full length LGC-50.

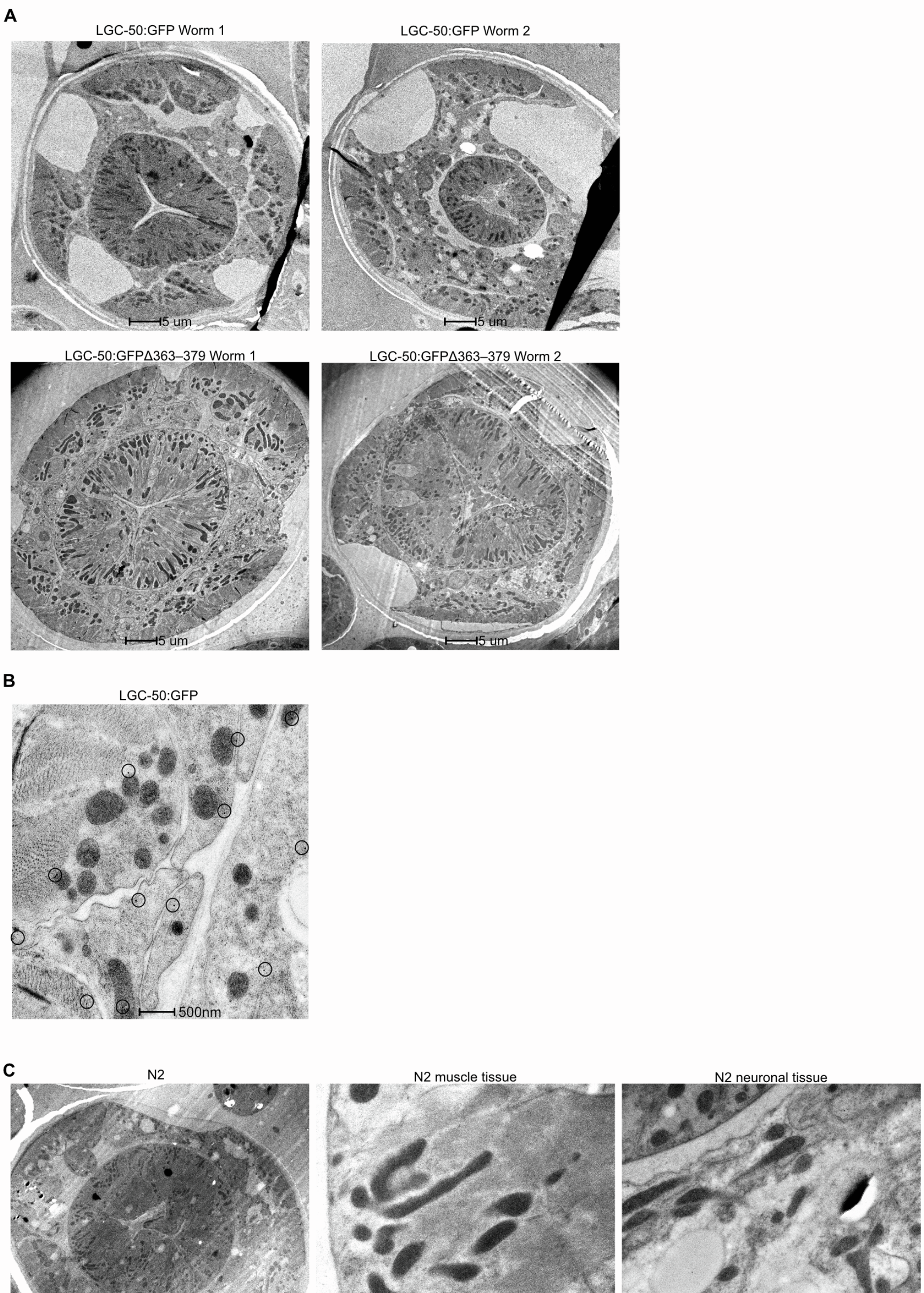

Supporting Figure 2. **Overview of sections used for EM imaging.** A. Overview of worm sections where high-resolution micrographs were acquired. B. Micrograph example of LGC-50::GFP with immuno-gold labelling. C. EM images from N2 worms after gold labelling, at different magnifications and cellular areas. Very little to no gold particles were found in these sections.

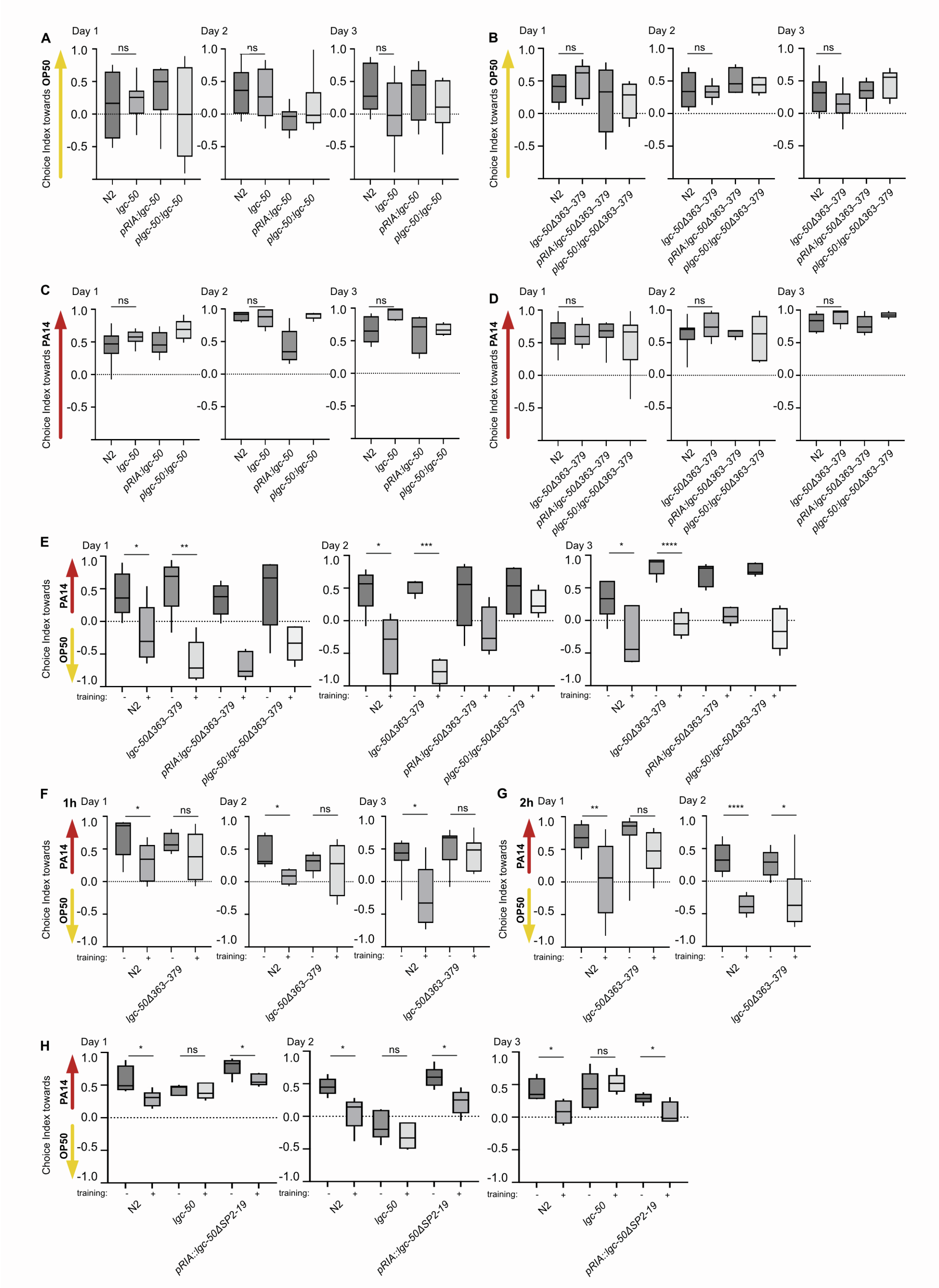

Supporting Figure 3. Individual chemotaxis data from each assay day. All behavioural tests were done over three independent days to confirm that the day-to-day variability was low and that test conditions were reproducible. A-B. Choice index calculated for chemotaxis towards OP50 over three different days of experiments. C-D. Choice index calculated for chemotaxis towards PA14 over three different days of experiments. E. Data from three different days for two-

choice aversive learning test. F-G. Data from three different days for two-choice aversive learning test with a 1- or 2-hour incubation time after training on PA14. H. Data from three different days for two-choice aversive learning test in signal peptide truncated *lgc-50*. *P < 0.05. ***P < 0.001, ****P < 0.0001, ns, no significant difference, calculated by Welch's t-test, data are shown as mean ± SEM.

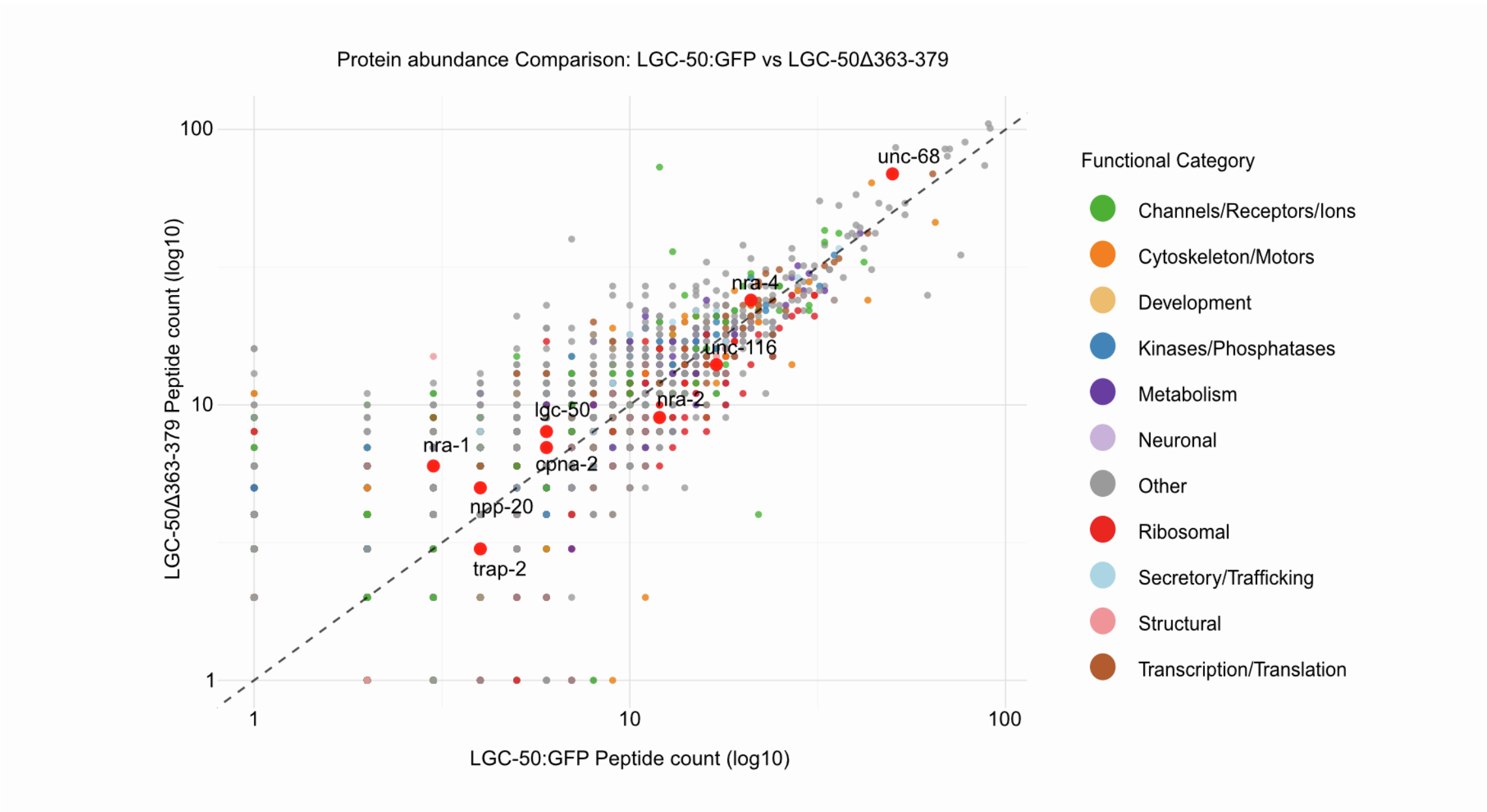

Supporting Figure 4. Protein abundance is samples used for LC-MS analysis. Protein abundance measured by LC-MS after immunoprecipitation of full-length LGC-50::GFP and LGC-50::GFPΔ363-379.

	LGC-50:GFP		LGC-50:GFPΔ363–379	
**Cellular area**	**Worm 1**	**Worm 2**	**Worm 1**	**Worm 2**
Cuticle	43	34	37	25
Internal empty zone	21	56	12	12
Internal muscle	226	138	277	337
Internal neuron	37	85	82	90
Membrane internal neuronal	8	11	3	5
Membrane muscle	49	60	100	51
Plasma membrane neuronal	36	56	39	25
Outside	30	81	21	60
**TOTAL**	450	521	571	605

Supporting Table 1. Gold particles identified with immuno-EM per worm sample and area across different tissues and cellular structures.
